# Assessing the Acceptability and Effectiveness of Mobile-Based Physical Activity Interventions for Midlife Women During Menopause: Systematic Review of the Literature

**DOI:** 10.2196/40271

**Published:** 2022-12-09

**Authors:** Ghada AlSwayied, Haoyue Guo, Tasmin Rookes, Rachael Frost, Fiona L Hamilton

**Affiliations:** 1 UCL Research Department of Primary Care and Population Health University College London London United Kingdom; 2 Department of Community Health Sciences King Saud University Riyadh Saudi Arabia

**Keywords:** mobile app, mobile health, mHealth, smartphone, smartphone apps, physical activity, exercise, midlife women, menopause, menopausal symptoms, behavior change, women’s health, wearable, activity tracker, effectiveness, acceptability, review, meta-analysis, mobile phone

## Abstract

**Background:**

Midlife women with menopausal symptoms are less likely to meet the recommended level of physical activity (PA). Promoting PA among women in midlife could reduce their risk of cardiovascular diseases and perhaps improve menopausal symptoms. Mobile PA interventions in the form of smartphone apps and wearable activity trackers can potentially encourage users to increase PA levels and address time and resource barriers to PA. However, evidence on the acceptability and effectiveness of these interventions among midlife women is unclear.

**Objective:**

This systematic review evaluated the effectiveness, acceptability, and active behavior change techniques (BCTs) of mobile PA technologies among midlife menopausal women.

**Methods:**

A mixed methods systematic review of qualitative and quantitative studies was conducted. MEDLINE (Ovid), Embase, Scopus, CINAHL, Web of Science, SPORTDiscus, CENTRAL, PsycINFO, and the ProQuest Sports Medicine and Education Index were systematically searched. Studies were selected and screened according to predetermined eligibility criteria. In total, 2 reviewers independently assessed the risk of bias using the Mixed Methods Appraisal Tool and completed BCT mapping of the included interventions using the BCT Taxonomy v1.

**Results:**

A total of 12 studies were included in this review. Overall risk of bias was “Moderate to high” in 58% (7/12) of the included studies and “low” in 42% (5/12) of the studies. Of the 12 studies, 7 (58%) assessed changes in PA levels. The pooled effect size of 2 randomized controlled trials resulted in a small to moderate increase in moderate to vigorous PA of approximately 61.36 weekly minutes among midlife women, at least in the short term (95% CI 17.70-105.01; P=.006). Although a meta-analysis was not feasible because of heterogeneity, positive improvements were also found in a range of menopause-related outcomes such as weight reduction, anxiety management, sleep quality, and menopause-related quality of life. Midlife women perceived mobile PA interventions to be acceptable and potentially helpful in increasing PA and daily steps. The average number of BCTs per mobile PA intervention was 8.8 (range 4-13) according to the BCT Taxonomy v1. “Self-monitoring of behaviour,” “Biofeedback,” and “Goal setting (behaviour)” were the most frequently described BCTs across the included interventions.

**Conclusions:**

This review demonstrated that mobile PA interventions in the form of smartphone apps and wearable trackers are potentially effective for small to moderate increases in moderate to vigorous PA among midlife women with menopausal symptoms. Although menopause is a natural condition affecting half the population worldwide, there is a substantial lack of evidence to support the acceptability and effectiveness of mobile PA interventions on menopause-related outcomes, which needs further investigation.

**Trial Registration:**

PROSPERO CRD42021273062; https://www.crd.york.ac.uk/prospero/display_record.php?RecordID=273062

## Introduction

### Background

Participation in regular physical activity (PA) confers clinically significant improvements in musculoskeletal, functional, and mental health–related outcomes, with an extensive evidence base on maintaining energy balance, lowering the risk of cardiovascular and metabolic diseases, and improving overall quality of life (QoL) [1-4].

Midlife women undergoing menopause tend to have a more noticeable decline in PA levels, being more physically inactive than men across most countries [5,6]. In England, only 23% and 21% of women aged 45 to 54 years and 55 to 64 years, respectively, met the National Health Service aerobic and muscle-strengthening guidelines recommended for adults (aged 19-64 years) [7,8]. The UK National Health Service guidelines for PA recommend that adults aged 19 to 64 years take part in a minimum of 150 minutes of moderate to vigorous PA (MVPA), 75 minutes of vigorous activity per week, or an equivalent combination of both alongside muscle-strengthening activities (eg, body and weight lifting, yoga, and Pilates) twice a week [9]. Research suggests that a reduction in PA levels parallels the drop in estrogen during the menopausal transition, a factor that may contribute to decreased PA and the shift to more sedentary behavior among midlife women [10-12].

Midlife is also a period when the risk of chronic diseases increases, potentially because of the cumulative effects of unhealthy lifestyle behaviors [13] and, most directly, as a result of menopause-associated weight gain and increased risk of abdominal obesity [14-16]. During the menopause transition, women may experience an array of bothersome symptoms that may overlap or have a cascade effect, with hot flushes, night sweats, and vaginal dryness most frequently reported [17]. Other psychosocial and physical complaints include weight gain, sleep disturbances, mood swings, anxiety, fatigue, joint aches, sexual dysfunction, heart palpitations, and deterioration of QoL [18]. Increasing PA levels may reduce menopausal symptoms and improve QoL [19-22]. Evidence is currently mixed [23-25], but there are plausible biological mechanisms by which PA can alleviate vasomotor symptoms, for instance, by releasing neuroendocrine substances (eg, cortisol) that are involved in stress and thermoregulatory body responses. PA may also attenuate weight gain influenced by menopausal transition and aging, as well as other physical and psychological symptoms such as body pain, fatigue, poor sleep, and depression [5].

The use of mobile phone–based interventions may potentially encourage midlife women to increase PA. Mobile PA technology is defined as the use of wireless devices such as smartphones, tablets, wearable activity trackers (WATs), and PDAs to promote PA and provide a means for real-time monitoring [26,27]. Apps that run on mobile platforms typically form part of these interventions. In this review, we adopted an operating definition of mobile-based PA interventions by referring to *the use of mobile app technology delivered through smartphones or WATs connected to partnering phone apps (eg, smartwatches or Fitbit) that can gather data and track progress remotely, with the aim of increasing PA participation in any form: aerobic (cardiovascular), resistance, endurance, or stretching exercise*.

Compared with men, women are more likely to use smartphones and health apps daily [28], and 83% of adults aged 55 to 64 years owned a smartphone in 2021 [29]. Moreover, women may particularly favor mobile-based interventions that use flexible delivery modes as a motivator to overcome the risk of not allocating sufficient time to be physically active [30-32]. Unlike in-person training programs, mobile PA interventions may encourage women to overcome physical barriers (ie, lack of time because of multiple responsibilities [33-36]) and feelings of stigma, social discomfort, and self-consciousness linked with participation in group-based PA programs and gym attendance [35,37], for example, a fear of being judged for decreasing abilities [37].

The global market of PA apps was valued at US $1.1 billion in 2021, with a 46% increase since May 2020 in global downloads of fitness and health apps [38]. In 2017, there were >325,000 commercially available health and fitness apps on the market [39]; approximately 30% of them targeted PA [38]. Emerging evidence indicates the potential of these apps to promote PA uptake [40] even among older adults, contributing to healthy aging [41-43]. However, despite the popularity of PA apps, the published evidence of their effectiveness from recent systematic reviews in adults shows positive but mostly nonsignificant effects [44-47].

Incorporating behavior change techniques (BCTs) and theories in developing and implementing such mobile-based interventions is an essential ingredient to ensure their acceptability and effectiveness. Goal-Setting Theory and Social Cognitive Theory (SCT) argue that, for a behavior change to occur, goals should be specific, learning-orientated, attainable in the short term but sufficiently challenging, and linked to a longer-term goal [48,49]. Regardless, many PA apps on the market have limited BCTs, for example, the ability to be tailored to users’ needs and characteristics [40,50]. Recently, several content analyses have been conducted to determine the active ingredients of commercially available consumer-facing PA apps using the comprehensive BCT Taxonomy v1 (BCTTv1) [51]. Of the 93 BCTs in the taxonomy, Middelweerd et al [52] and Bondaronek et al [53] found that, on average, only 5 and 7 BCTs were used among 64 and 65 commercially available PA apps reviewed, respectively.

Furthermore, the key to successful digital behavior change interventions is potentially determined by the acceptability of the intervention and the level of motivation and user engagement [54,55]. Acceptability is a multifaceted construct that reflects how individuals consider an intervention to be appropriate based on anticipated and experienced responses to it.

### Gaps in the Current Knowledge

Although the literature on the impact of mobile PA apps and WATs on adult and older adult populations is growing, to date, midlife women are largely neglected. In total, 2 pretest-posttest studies indicate that app- and web-based interventions may increase PA in this population [56,57] and may have advantages over conventional PA interventions [58]. However, to our knowledge, no review has synthesized current evidence on the contribution of mobile PA technology to changes in PA and menopause-related health outcomes among midlife women.

### Aim

This mixed methods systematic review aimed to investigate and consolidate the existing evidence on the effectiveness, acceptability, and active behavior change components of mobile technologies for PA in midlife menopausal women. The following review questions were addressed: (1) How effective are mobile PA interventions in increasing PA levels in midlife women? (2) How effective are mobile PA interventions in improving menopause-related symptoms in midlife women? (3) How acceptable are mobile PA interventions for midlife women with menopausal symptoms? (4) Which BCTs are used across mobile PA interventions for midlife women during menopause?

## Methods

### Design

A mixed methods systematic review of qualitative and quantitative studies was conducted following the 2020 PRISMA (Preferred Reporting Items for Systematic Reviews and Meta-Analyses) guidelines [59]. The protocol was registered in PROSPERO (CRD42021273062).

### Information Sources and Search Strategy

Nine electronic databases—MEDLINE (Ovid), Embase, Scopus, CINAHL, Web of Science, SPORTDiscus, CENTRAL, PsycINFO, and the ProQuest Sports Medicine and Education Index—were systematically searched from January 1, 2007 (the year the first mobile app emerged on the market), to August 2021, updated in February 2022. Subsequently, a further forward and backward citation search and screening of reference lists of the included papers were used to detect any additional relevant studies. If the full text could not be found through searches, the corresponding authors of potentially relevant studies were contacted via email to request access to full-text papers or inquire about ongoing trial protocols.

The search strategy was developed and refined iteratively based on expert consultation with a systematic search librarian at University College London. The search strategy combined three key terms—“mobile digital interventions” AND “physical activity” AND “menopausal women”—including synonyms and components (eg, “mHealth,” “wearables,” “mobile apps,” “Fitbit,” and “smartwatch”). The search strategy was adapted for each database using tailored syntax, Boolean operators, and Medical Subject Heading terms. The full details of the search strategy can be found in Multimedia Appendix 1.

Systematic database searching was supplemented with gray literature searches using the Google Scholar and Google search engines. Search results were sorted by relevance, and the first 20 pages (approximately 200 results) were reviewed. However, no additional papers that met the eligibility criteria were identified through this process, and so gray literature was excluded.

### Eligibility Criteria

The inclusion criteria were developed based on the Participant, Intervention, Control, and Outcome structure (Textbox 1). Studies of any design comprising quantitative (randomized, nonrandomized, and pretest-posttest studies), qualitative, and mixed methods primary research were all included. Studies that assessed the measurement properties or algorithm performance of digital interventions with no health or behavior change outcomes measured were excluded. Commentaries, conference abstracts, editorials, reviews, registered protocols with no results published, theses, books, and studies not providing an explicit research methodology were excluded.

**Inclusion and exclusion criteria (Participant, Intervention, Control, and Outcome structure).**

**Participant**

- Inclusion criteria:
  Midlife women either defined by age range (40-64 years) or menopause stage (perimenopause, menopause, and postmenopause) and experiencing at least one menopausal symptom such as hot flushes, night sweats, weight gain, sleep problems, vaginal dryness, mood swings, or anxiety
  No restrictions on geographical location, ethnicity, or presence of comorbidities or risk factors, including studies targeting survivors of breast cancer in menopause age (40-64 years) owing to the general age-related needs and preferences
- Exclusion criteria:
  Older or late postmenopausal women (aged >65 years) as they may have different views and concerns with regard to mobile physical activity (PA) technologies
  Studies targeting men or the middle-aged population in general if extracting gender-specific outcomes is not possible
  Midlife women undergoing hormonal replacement therapy (HRT), which can act as an active treatment for menopausal symptoms
  Women with premature ovarian insufficiency as HRT is likely to be prescribed to inhibit the development of osteoporosis, atherosclerosis, cardiovascular diseases, dementia, and mortality in younger ages [60,61]

**Intervention**

- Inclusion criteria:
  Mobile-based PA interventions functioning as workout fitness programs, step count, self-monitors, walking-route trackers, or social networking site fitness interventions
  Either stand-alone mobile apps or apps paired with wearable activity trackers (WATs)
  No restriction on the dose or duration of app use or length of the intervention and whether the interventions were supervised or self-delivered
  Apps targeting multiple lifestyle behaviors only if PA outcome data were extracted independently
- Exclusion criteria:
  Interventions based on traditional prompts (eg, email, phone calls, or SMS text messaging)
  Traditional or electronic activity trackers (ie, pedometers or ActiGraph accelerometer–based interventions) unless used in conjunction with an app or as an objective measure of PA outcomes for an app
  Passive mobile interventions where users did not have to log in, engage, or monitor PA themselves, such as software to be accessible only by clinicians and researchers

**Control**

- Inclusion criteria:
  If applicable, control groups administering either no intervention or no mobile-based intervention, such as printed materials or traditional pedometers where users could not interact or receive instant feedback
- Exclusion criteria:
  Any app-based controls

**Outcome**

- Inclusion criteria:
  Changes in the frequency, intensity, or duration of PA reported in any form (eg, weekly minutes of moderate to vigorous PA, daily steps, or energy expenditure) measured using either self-reported or objective measures (ie, accelerometers)
  Changes in the frequency or severity of any common menopause-related symptoms (eg, vasomotor, sleep disturbance, weight gain, and depression) measured using validated scales and generic or menopause-specific quality of life measured using validated scales (eg, the bothersome scale, the Greene Climacteric Scale, or generic or menopause-specific scales such as the Menopause-Specific Quality of Life Questionnaire)
  Acceptability data through qualitative methods with respect to user satisfaction and experiences, perceived usefulness, usability, and intention to use [62] as well as engagement and interaction with the app, including quantitative data on app or WAT use and compliance
- Exclusion criteria:
  Studies that did not report the measurement of at least one of the primary or secondary outcomes of interest specified in the review protocol (eg, measurement of cancer-specific outcomes only)

### Screening and Selection Procedure

After removing duplicates using EndNote (version 20; Clarivate Analytics) [63], the first reviewer (GS) screened all titles and abstracts in the first round and then reviewed the full text of potentially relevant or unclear articles against the eligibility criteria using Rayyan (Rayyan Systems, Inc) [64]. A second reviewer (HG) independently reviewed the first 20.83% (215/1032) of the retrieved records, alphabetically sorted by title, and tested them against the eligibility criteria. The percentage of agreement between the reviewers (GS and HG) was 92%, showing substantial interrater reliability (Multimedia Appendix 2). Disagreements between the reviewers were resolved through discussion and, where necessary, consultation with FH and RF.

### Data Extraction

GS and HG independently extracted data using an adapted data extraction form following the Cochrane Collaboration standardized data extraction templates for quantitative and qualitative studies [65]. The following data were extracted: study characteristics (publication year, authors, and country); study type and aims; participant characteristics and context (sample size, mean age, and menopause stage if available); a description of the interventions as recommended by the Template for Intervention Description and Replication checklist [66], including content, mode of delivery, features, duration, intensity, and theoretical contribution; outcomes measured on the overall effectiveness of mobile PA technology on any menopause-relevant outcomes and PA outcomes as well as the acceptability, user engagement, and adherence to the intervention; and control group treatment (if applicable). In the case of registered or ongoing trials and protocols, we attempted to contact the corresponding authors via email to seek additional unpublished information where applicable (4 were contacted and 2 responded).

### Quality Assessment

Two authors (GS and HG) assessed the methodological quality of each included study independently using the Mixed Methods Appraisal Tool [67] and discussed their assessments to achieve consensus. In this review, we used a star rating system as the Mixed Methods Appraisal Tool has no established quality threshold for inclusion and classification of overall risk of bias [68]. Studies were rated as “low risk of bias” if they obtained stars in up to four domains and as “moderate-to-high risk of bias” when they were awarded stars on ≤3 domains. Studies were not excluded based on critical appraisal given the infancy of research in this area. However, studies with moderate to high risk of bias were reported with caution.

### BCT Coding

For the included studies with actual mobile PA technology (9/12, 75%), 2 reviewers trained in BCT coding (GS and TR) independently coded all PA interventions in both the intervention and (active) control groups using the BCTTv1 [51]. Published descriptions and supplementary materials, if available, were reviewed in full. All discrepancies between the 2 initial coders were resolved through discussion until agreement was achieved. If necessary, the third and fourth reviewers (FH and RF) were also consulted to mediate an agreement. The average number and type of BCTs used were mapped for each studied intervention.

### Data Synthesis

Narrative synthesis following the guidelines by Popay et al [69] was used for this review because of the heterogeneity of interventions, populations, and outcomes measured. The approach by Popay et al [70] allows for transparency of narrative synthesis by interpreting evidence from different methodologies.

Quantitative data were tabulated, with textual descriptions applied to draw a preliminary synthesis of the findings. Qualitative data were coded inductively in NVivo (version 12; QSR International) using thematic synthesis [71], and analytical themes were generated. In this review, we drew on the technology acceptance model (TAM) [72] to guide the analysis of qualitative data. The TAM suggests that an individual’s intention to use technology is based on two key factors: perceived usefulness, which refers to a user’s beliefs that engaging with the app improves their PA performance, and perceived ease of use, which refers to the perception that using the app requires minimal effort [72]. Although the TAM assumes that acceptability does not change over the life cycle of a digital intervention, it is widely used and has been shown to be robust in several empirical studies [73].

### Meta-analysis

A meta-analysis was conducted for randomized controlled trials (RCTs) only using RevMan (The Cochrane Collaboration) [74] where sufficient studies were available for an outcome. Pooling change scores within and between groups is not recommended [65]; therefore, pre-post studies were not meta-analyzed. Effect sizes were calculated using the absolute mean difference and associated 95% CI between the final values observed for the experimental and control groups. A random-effects model was used to allow for between-study variability. Heterogeneity was quantified using *I*^2^. Owing to the small number of included studies, tests for asymmetry and publication bias could not be conducted.

## Results

### Study Selection

**Figure 1:**
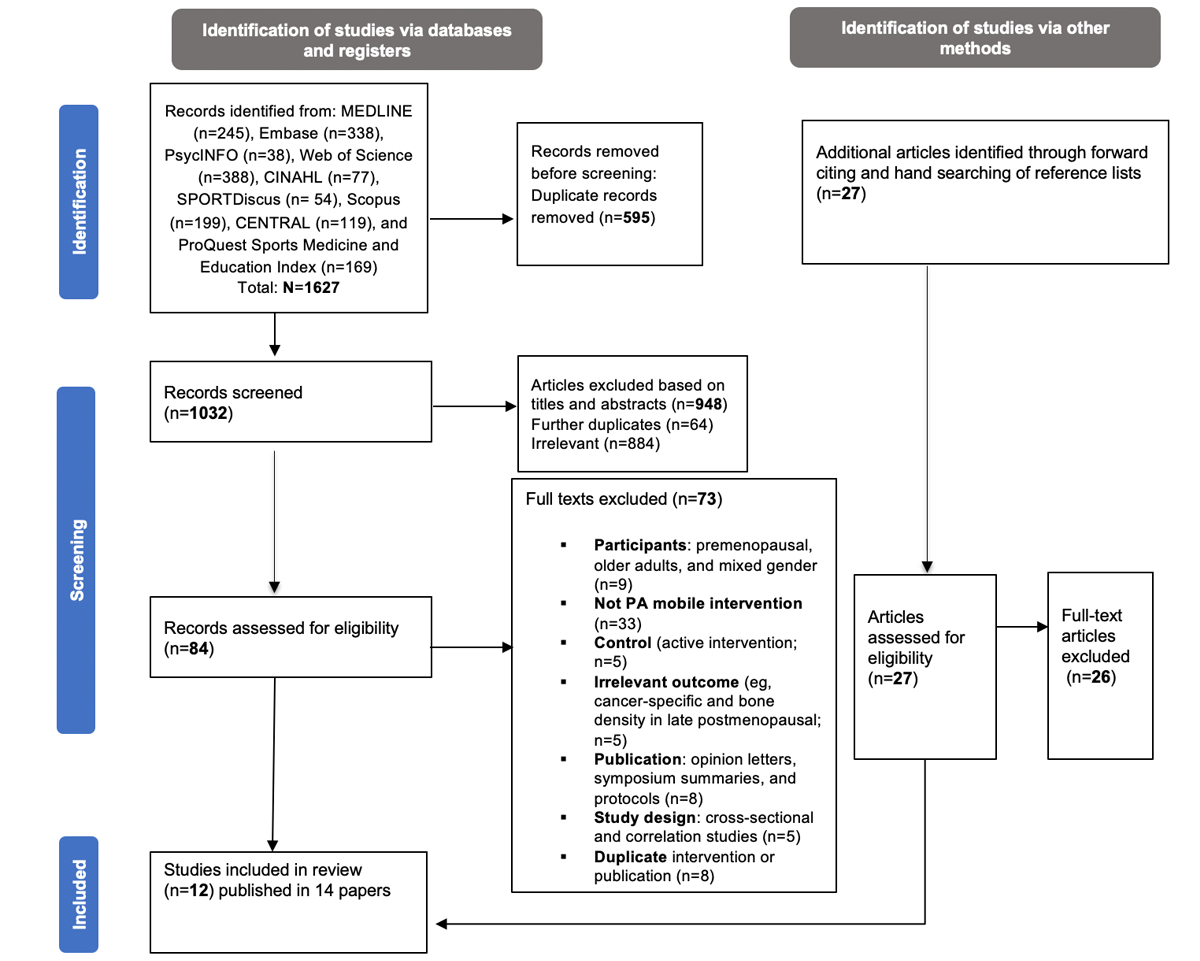
Study selection flow diagram based on the PRISMA (Preferred Reporting Items for Systematic Reviews and Meta-Analyses) statement. PA: physical activity.

The study selection process is summarized in Figure 1 using the PRISMA flow diagram. Of 1627 records identified in addition to 27 potentially relevant records, citation tracking, and reference list screening, 12 studies (0.73%) published in 14 papers were included in the final review synthesis [56,75-85]. Reasons for exclusion are presented in Multimedia Appendix 3, mainly the absence of mobile PA technology, followed by irrelevant age groups.

### Characteristics of the Included Studies

See Table 1 for characteristics of the included studies (N=12). The studies reflected cross-disciplinary research and different stages of intervention development and evaluation. The studies were conducted in the United States (6/12, 50%), Australia (2/12, 17%), South Korea (2/12, 17%), Italy (1/12, 8%), and Iran (1/12, 8%). In total, 75% (9/12) of the studies were published in the last 5 years.

The included studies were a mix of quantitative (7/12, 58%), qualitative (4/12, 33%), and mixed methods (1/12, 8%) studies. The sample sizes ranged from 8 [77] to 83 participants [83]. Of the 12 studies, 4 (33%) were pilot RCTs, of which 1 (25%) had an active control arm [78] and 3 (75%) had waitlist or no-intervention control groups [76,81,83]. In total, 25% (3/12) of the studies were pretest-posttest studies [56,79,84]. The quantitative study duration varied from 1 [76] to 6 months [83]. Qualitative studies (4/12, 33%) included a semistructured focus group (1/4, 25%), semistructured interviews (1/4, 25%), and participatory design (2/4, 50%).

The participants were midlife women with an average age of 57.6 (SD 4.026) years. Most of the included studies (10/12, 83%) recruited women based on age range, followed by menopause stage, with only 17% (2/12) of the studies [75,77] identifying participants based on the experience of menopausal symptoms. The studied women were culturally diverse; 17% (2/12) of the studies [56,81] targeted African American women, and 8% (1/12) targeted [85] Korean-Chinese migrants. The included participants were heterogeneous concerning health conditions and the presence of chronic diseases. A total of 42% (5/12) of the studies limited recruitment to inactive (ie, ≤60 minutes per week of MVPA) and overweight (mean BMI 29.2, SD 3.5 kg/m^2^) or obese (mean BMI 33.9, SD 5.9 kg/m^2^) women [56,78,79,82,83]. In total, 25% (3/12) of the studies were based on midlife women diagnosed with breast cancer [80,81,83], and 8% (1/12) recruited postmenopausal women from cardiology clinics [84].

**Table 1:** Characteristics of all the included quantitative and qualitative studies and studied populations (N=12).

Author, year	Country	Study design (duration)	Sample size, N	Retention rate at follow-up	Age (years)	Menopausal stage	Experience of menopausal symptoms	Eligibility for recruitment	Overall risk of bias
Cadmus-Bertram et al [78], 2016	United States	RCT^a^ (16 weeks)	51	96% (49/51)	Mean 60 (SD 7.1)	Postmenopausal	Not given	Inactive, overweight (mean BMI 29.2, SD 3.5 kg/m^2^)	Low risk
Valle et al [81], 2017	United States	RCT (6 months)	35	94.3% (33/35)	Mean 53 (SD 9.1)	80% postmenopausal	Not given	African American, obese (mean BMI 33.9, SD 5.9 kg/m^2^); diagnosed with breast cancer in the last 10 years	Low risk
Lynch et al [83], 2019	Australia	RCT (12 weeks)	83	96% (80/83)	Mean 61.6 (SD 76.4)	Postmenopausal	Not given	Inactive, overweight (mean BMI 29, SD 6.0 kg/m^2^); diagnosed with breast cancer and had completed treatment	Low risk
Kashfi et al [76], 2021	Iran	RCT (1 month)	54	Not reported	Mean 53.9 (SD 4.03)	Menopausal and postmenopausal	Not given	Aged between 45 and 60 years and at least 1 year after the last menstruation; no hormone therapy over the past 6 months	Moderate to high risk
Butryn et al [79], 2016	United States	Pre-post (6 months)	36	78% (28/36)	Mean 54 (SD 7.18)	Not identified	Not given	Inactive, aged between 40 and 65 years	Moderate to high risk
Sengupta et al [84], 2020	United States	Pre-post (12 weeks)	10	80% (8/10)	Mean 64 (SD 6.0)	Not identified	Not given	Aged ≥50 years, recruited from cardiology clinics	Moderate to high risk
Joseph et al [56], 2021	United States	Pre-post (4 months)	20	80% (16/20)	Mean 56.2 (SD 4.3)	Not identified	Not given	African American, aged 50 to 65 years, inactive (≤60 minutes per week of MVPA^b^), and BMI of 40.0 (SD 8.6) kg/m^2^	Moderate to high risk
Lee et al [75], 2015^c^	South Korea	Qualitative; semistructured interviews	9	N/A^d^	Range 45 to 60	Perimenopausal	Experiencing menopause symptoms	Aged between 45 and 60 years, experiencing or having experienced menopausal symptoms within the last 5 years	Low risk
Nguyen et al [80], 2017	Australia	Qualitative; focus group	14	N/A	Mean 58.6	Postmenopausal	Not given	Active and inactive; diagnosed with breast cancer	Low risk
Senette et al [82], 2018^c^	Italy	Qualitative; participatory design focus group	26	N/A	Range 45 to 60	Perimenopausal	Not given	Aged between 45 and 60 years, 18.5<BMI<30, and absence of chronic diseases	Moderate to high risk
Backonja et al [77], 2021^c^	United States	Qualitative; participatory design focus group	8	N/A	Range 40 to 64	Perimenopausal and early postmenopausal	Experiencing menopause symptoms	Aged 40 to 64 years	Moderate to high risk
Kim et al [85], 2020	South Korea	Mixed methods; focus group and validity pilot test	Focus group: 16; pilot study: 12	N/A	Range 40 to 65	Not given	Not given	Korean-Chinese; aged 40 to 65 years; full-time workers for the last 6 months	Moderate to high risk

^a^RCT: randomized controlled trial.

^b^MVPA: moderate to vigorous physical activity.

^c^Preclinical studies of IT research (menopause informatics).

^d^N/A: not applicable.

### Quality Assessment of the Included Studies

The overall risk of bias was “Moderate to high” in 58% (7/12) of the included studies and “low” in 42% (5/12) of the studies**.** All RCT groups (4/12, 33%) were comparable at baseline, whereas randomization was adequately performed and sufficiently reported in 75% (3/4) of these studies. Owing to the nature of mobile PA interventions, participant and assessor blinding could not be achieved in any study. There was poor reporting of a WhatsApp-based intervention and PA outcomes [76].

None of the 25% (3/12) of pre-post studies [56,79,84] accounted for confounders in the design and analysis, reducing the confidence in the observed effects (poor quality overall with high risk of bias). The included pre-post studies (3/12, 25%) had very small sample sizes and low recruitment rates; for instance, Sengupta et al [84] recruited 10 midlife women, and only 8 completed the 12-week follow-up. Similarly, Joseph et al [56] reported a low recruitment rate of 22% with a small sample size of 20.

There was better reporting across the qualitative studies except for reflexivity and the authors’ positions. A lack of data reporting and integration was observed in the mixed methods study design by Kim et al [85]. The risk of bias scoring system is presented in Tables 2-5.

**Table 2:** Summary of Mixed Methods Appraisal Tool quality assessment—risk of bias of the included randomized controlled trials (RCTs).

RCT, year	Randomization appropriately performed	Groups comparable at baseline	Complete outcome data	Outcome assessors blinded to the intervention	Participants adhered to the assigned intervention	Risk of bias score^a^
Cadmus-Bertram et al [78], 2016	★^b^	★	★	0^c^	★	Low
Valle et al [81], 2017	★	★	★	0	★	Low
Lynch et al [83], 2019	★	★	★	0	★	Low
Kashfi et al [76], 2021	0	★	★	0	—^d^	Moderate to high

^a^Overall risk of bias scores were assessed by 2 independent reviewers and classified into low risk and moderate to high risk. Low risk of bias: ≥4 stars; moderate to high risk of bias: ≤3 stars.

^b^Met the criterion.

^c^Failed to meet the criterion.

^d^Insufficient information given to decide.

**Table 3:** Summary of Mixed Methods Appraisal Tool quality assessment—risk of bias of the included pre-post studies.

Pre-post study, year	Representativeness of the target population	Measurements appropriate for outcome and intervention	Complete outcome data	Confounders accounted for in the design and analysis	Intervention and exposure happened as intended	Risk of bias score^a^
Butryn et al [79], 2016	0^b^	★^c^	★	0	★	Moderate to high
Sengupta et al [84], 2020	0	★	★	0	★	Moderate to high
Joseph et al [56], 2021	0	★	★	0	★	Moderate to high

^a^Overall risk of bias scores were assessed by 2 independent reviewers and classified into low risk and moderate to high risk. Low risk of bias: ≥4 stars; moderate to high risk of bias: ≤3 stars.

^b^Failed to meet the criterion.

^c^Met the criterion.

**Table 4:** Summary of Mixed Methods Appraisal Tool quality assessment—risk of bias of the included mixed methods study.

Mixed methods study, year	Adequate rationale for using a mixed methods design	Integration of different components of the study	Adequate interpretation of outputs of the integration	Inconsistencies between qualitative and quantitative data	Different components adhered to the quality criteria of the methods involved	Risk of bias score^a^
Kim et al [85], 2020	—^b^	★^c^	★	0^d^	★	Moderate to high

^a^Overall risk of bias scores were assessed by 2 independent reviewers and classified into low risk and moderate to high risk. Low risk of bias: ≥4 stars; moderate to high risk of bias: ≤3 stars.

^b^Insufficient information given to decide.

^c^Met the criterion.

^d^Failed to meet the criterion.

**Table 5:** Summary of Mixed Methods Appraisal Tool quality assessment—risk of bias of the included qualitative and mixed-methods studies.

Qualitative study, year	Appropriate to answer the research question	Adequate qualitative data collection methods used	Findings adequately derived from the data	Sufficient interpretation of results	Coherence in data collection, analysis, and interpretation	Risk of bias score^a^
Lee et al [75], 2015	★^b^	★	★	★	—^c^	Low
Nguyen et al [80], 2017	★	★	★	★	★	Low
Senette et al [82], 2018	★	★	—	★	—	Moderate to high
Backonja et al [77], 2021	★	★	—	—	—	Moderate to high

^a^Overall risk of bias scores were assessed by 2 independent reviewers and classified into low risk and moderate to high risk. Low risk of bias: ≥4 stars; moderate to high risk of bias: ≤3 stars.

^b^Met the criterion.

^c^Insufficient information given to decide.

### Characteristics of the Included Interventions

All 12 studies included at least one form of mobile-enabled PA intervention, either as solo mobile apps or web-based applications or paired apps with other sensor-based activity trackers (ie, wearables). The intervention components included in-person training and behavior modification sessions [79,81,83], traditional SMS text messaging or follow-up calls [78,81,83,85], and educational pamphlets [76,83].

See Table 6 for the characteristics of the intervention types, embedded BCTs, and outcomes measured for the interventional studies (9/12, 75%). In total, 78% (7/9) used wearable devices to track activity paired with an app, including Fitbit (4/9, 44%) [56,78-80] and Garmin (2/9, 22%) trackers [80,83] and a tailored smartwatch paired with the HerBeat app (1/9, 11%) [84]. All studies were based on apps designed to promote PA except for 11% (1/9) of the studies [76], which used a WhatsApp-based PA intervention. Control groups included a basic step-counting pedometer without feedback [78] and a waitlist [81,83].

**Table 6:** Characteristics of the included mobile physical activity (PA) interventions, coded behavior change techniques (BCTs), and outcomes measured (N=9).

Author, year	Mobile PA technology	Control group (if applicable)	Duration	BCTs	Theoretical contribution	Outcomes measured
Cadmus-Bertram et al [78], 2016	Fitbit-based PA intervention	Basic step-counting pedometer+printed materials	16 weeks	Intervention group: 1.1 Goal setting (behavior) 1.4 Action planning 1.5 Review behavior goals 2.2 Feedback on behavior 2.3 Self-monitoring of behavior 2.6 Biofeedback Control group: 1.1 Goal setting (behavior) 1.2 Problem solving 1.4 Action planning 2.3 Self-monitoring of behavior	The CALO-RE^a^ framework, known as a comprehensive and standardized protocol for the identification, reporting, and appraisal of behavior change interventions for health behaviors, including PA [86]	MVPA^b^ (minutes per week) and increased steps per day using ActiGraph GT3X+ accelerometerc Height and weight measured using standard procedures and BMI^c^
Valle et al [81], 2017	Self-weighing and activity tracker mobile intervention	Waiting list	6 months	2.6 Biofeedback 2.3 Self-monitoring of behavior 1.2 Problem solving 2.2 Feedback on behavior 2.4 Self-monitoring of outcome of behavior 1.6 Discrepancy between behavior and goal 3.1 Social support (unspecified) 4.1 Instruction on how to perform the behavior 5.1 Information about health consequences 7.1 Prompts and cues 8.3 Habit formation 12.5 Adding objects to the environment	SRT^d^, a set of psychological subfunctions that must be mobilized for self-directed change [87]	Weight change, measured using BMI and waist circumferencec Energy expenditure (kcal per week), measured using the PAQ^e,c^
Lynch et al [83], 2019	Wearable activity monitor and app (Garmin)	Waiting list	12 weeks	2.6 Biofeedback 1.1 Goal setting (behavior) 1.2 Problem solving 1.5 Review behavior goals 2.3 Self-monitoring of behavior 2.2 Feedback on behavior 3.1 Social support (unspecified) 4.1 Instruction on how to perform the behavior 5.3 Information about social and environmental consequences 7.1 Prompts and cues	None	MVPA (minutes per week) using ActiGraph GT3X+^f^ Sedentary time, measured using an activPAL^f^ Sleep disturbance, measured by actigraphy and self-reported PSQI^g,f^
Kashfi et al [76], 2021	WhatsApp-based mobile intervention	No intervention	1 month	1.1 Goal setting (behavior) 1.2 Problem solving 1.4 Action planning 3.1 Social support (unspecified) 5.1 Information about health consequences 4.1 Instruction on how to perform the behavior 6.1 Demonstration of the behavior 7.1 Prompts and cues	None	QoL^h^ measured using self-reported MENQOL^i,f^
Butryn et al [79], 2016	Fitbit-based, blended PA intervention	Baseline	6 months	1.1 Goal setting (behavior) 1.2 Problem solving 1.6 Discrepancy between behavior and goal 2.2 Feedback on behavior 2.3 Self-monitoring of behavior 2.5 Monitoring of outcome of behavior without feedback 2.6 Biofeedback 3.1 Social support (unspecified) 6.2 Social comparison 9.1 Credible source	None	MVPA (minutes per week), measured using ActiGraph GT3X+^j^ Sedentary time^j^ Weight loss, measured using a standardized scale^j^
Sengupta et al [84], 2020	Smartwatch and smartphone app (HerBeat)	Baseline	12 weeks	1.1 Goal setting 1.4 Action planning 1.6 Discrepancy between behavior and goal 2.1 Monitoring of behavior by others without feedback 2.3 Self-monitoring of behavior 2.6 Biofeedback 5.3 Information about health consequences 7.1 Prompts and cues 10.4 Social reward	None	Change in PA using IPAQ-SF^k,l^ Exercise and dietary self-efficacy using the Exercise Condensed Survey^l^ Weight circumference and BMI^j^ Depressive symptoms, measured using the PHQ-9^m,j^ Perceived stress^l^
Joseph et al [56], 2021	Smart walk app and Fitbit	Baseline	4 months	1.1 Goal setting (behavior) 1.2 Problem solving 2.3 Self-monitoring of behavior 2.6 Biofeedback 3.1 Social support (unspecified) 4.1 Instruction on how to perform the behavior 5.3 Information about social and environmental consequences 6.1 Demonstration of the behavior	SCT^n^, proposes that people are driven not by inner forces but by external factors [88]	MVPA (minutes per week) using the 2-item Exercise Vital Sign Questionnaire^j^ Weekly estimated energy expenditure^j^ Changes in SCT mediators measured using self-reported questionnaires^j^, with unexpected decrease in self-efficacy for PA
Nguyen et al [80], 2017	WATs^o^ and paired apps: Fitbit One, Jawbone UP24, Garmin, Vivofit 2, Garmin Vivosmart, Garmin Vivoactive, and Polar A300	N/A^p^	4 weeks	2.3 Self-monitoring of behavior 2.6 Biofeedback 4.1 Instruction on how to perform the behavior 7.1 Prompts and cues	None	Preferences and experience of WATs to promote PA behavior change among postmenopausal women (generated themes)
Kim et al [85], 2020	Mobile-based living laboratory intervention Fitbit and mobile app	N/A	24 weeks	1.1 Goal setting (behavior) 1.2 Problem solving 1.5 Review behavior goals 2.3 Self-monitoring of behavior 2.6 Biofeedback 3.1 Social support (unspecified) 3.3 Social support (emotional) 5.1 Information about health consequences 6.1 Demonstration of the behavior 4.1 Instruction on how to perform the behavior 7.1 Prompts and cues 10.4 Social reward 15.1 Verbal persuasion about capability	SCT [88]	Involvement of middle-aged, migrant women in the development of a culturally appropriate, mobile intervention to promote PA Validity testing of the developed app based on content, interface design, and technology criteria using a 23-item self-reported smartphone app evaluation tool for health care

^a^CALO-RE: Coventry, Aberdeen, and London-Refined.

^b^MVPA: moderate to vigorous physical activity.

^c^No statistically significant difference between the groups (no evidence).

^d^SRT: self-regulation theory.

^e^PAQ: Paffenbarger Physical Activity Questionnaire.

^f^Statistically significant difference between the groups (significant evidence).

^g^PSQI: Pittsburgh Sleep Quality Index.

^h^QoL: quality of life.

^i^MENQOL: Menopause-Specific Quality of Life Questionnaire.

^j^Statistically significant difference from baseline (some supporting evidence).

^k^IPAQ-SF: International Physical Activity Questionnaire-Short Form.

^l^No statistically significant difference from baseline (no evidence).

^m^PHQ-9: Patient Health Questionnaire-9.

^n^SCT: Social Cognitive Theory.

^o^WAT: wearable activity tracker.

^p^N/A: not applicable.

### Effectiveness of Mobile PA Technologies in Menopausal Women

#### PA Behavior Change

**Figure 2:**
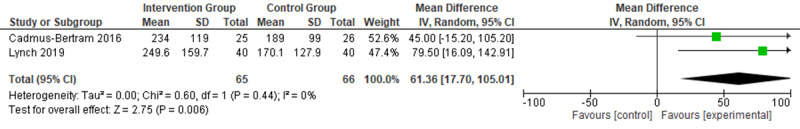
Meta-analysis results of between-group difference in moderate to vigorous physical activity (MVPA; mins per week) of the 2 included randomized controlled trials that reported MVPA measurements.

Change in PA was measured in 86% (6/7) of the included quantitative studies using MVPA (minutes per week), energy expenditure (kcal per week), or a number of daily steps. In total, 50% (2/4) of the RCTs (n=131) reported changes in MVPA (minutes per week) between the groups [78,83]. Compared with the control groups, the use of mobile-based PA interventions (wearables and their paired apps) significantly improved MVPA by 61.36 minutes per week (95% CI 17.70-105.01; P=.006) after 16 weeks of intervention. There was no evidence of statistical heterogeneity (*I*^2^=0%; P=.44); however, the CIs were very wide, and the sample sizes were small, suggesting that this should be interpreted with caution (Figure 2).

The findings of other studies were mixed. The RCT by Valle et al [81] used the self-reported Paffenbarger Physical Activity Questionnaire (PPAQ) to measure energy expenditure (kcal per week) as a secondary outcome and found no statistical difference between the groups in PA over 6 months of follow-up. There was no reporting of quantitative analysis, and the authors did not respond to queries. In the 25% (3/12) of pre-post studies, MVPA (minutes per week) was measured using objective ActiGraph GT3X+ accelerometers, the International Physical Activity Questionnaire-Short Form (IPAQ-SF), and Exercise Vital Sign self-reported questionnaires [56,79,84]. Butryn et al [79] found a significant modest increase in MVPA from 63 minutes per week at baseline to 135 minutes per week after 6 months (P=.01). Similarly, Joseph et al [56] self-reported a significant increase in MVPA from 20 minutes per week at baseline to 50 minutes per week after 1 month of intervention (P<.001). Sengupta et al [84] found a moderate increase in PA from 35.6 minutes per day at baseline to 63.1 minutes per day at 3 months; however, this did not reach statistical significance.

#### Sedentary Time

Only 17% (2/12) of the studies [79,83] assessed the impact of mobile PA interventions on sedentary time. Lynch et al [83] measured sedentary behavior using an activPAL and found a moderate significant decrease of −36.6 minutes per day (95% CI −71.7 to −1.6) between the groups after 12 weeks of intervention (P=.01). Butryn et al [79] found a nonclinically significant decrease in sedentary time from 75.6 (SD 5.72) minutes per day to 73.2 (SD 5.81) minutes per day at the 6-month follow-up (P<.05).

#### Self-efficacy

Self-efficacy (SE) to exercise was measured in 67% (2/3) of the pre-post studies [56,84]. Neither found a significant positive effect on SE. Joseph et al [56] also measured other social cognitive mediators—self-regulation, behavioral capability, expectations, and social support—using self-reported questionnaires. Over the 4-month mobile PA intervention, the results showed significant improvements in other social cognitive mediators such as behavioral capability for PA (*r*=0.440; P=.004). However, unexpectedly, they found a decreased negative trend in exercise SE for PA (*r*=−0.364; P=.02) after 4 months of intervention [56]. The authors did not report any explanation for this unexpected decrease in SE.

### Menopause-Related Outcomes

The measures included were weight loss, sleep disturbance, mental health (perceived stress and depressive symptoms), and menopause-specific QoL.

#### Weight Loss

Changes in weight were assessed in 33% (4/12) of the studies [78,79,81,84]. BMI was measured by one 3-arm RCT [81]. Valle et al [81] reported a borderline significant marginal decrease in BMI of −0.4 kg/m^2^ (95% CI −1.7 to −0.1) over 6 months (P=.046) between the mobile-based technology intervention and control groups.

The findings for weight change were mixed, with nonclinically meaningful effects. Both RCTs [78,81] found no statistically significant difference between the intervention and control groups for weight, measured in kilograms, and median percent weight change (IQR). Cadmus-Bertram et al [78] found a nonstatistical difference of 0.06 between the web-based intervention and pedometer control groups after 16 weeks of intervention (P=.61). Similarly, Valle et al [81] reported nonstatistically significant weight loss over 6 months favoring the interventional group of both the PA tracker and self-weighing mobile intervention (P=.07) but not the self-weighing only intervention group (P=.36) compared with the control group. Owing to high heterogeneity and different reported outcomes of the 2 RCTs [78,81], a meta-analysis did not seem to be appropriate.

In total, 67% (2/3) of the pre-post studies measured change from baseline [79,84]. Butryn et al [79] found a statistically significant weight loss of 1.86 kg from baseline to the 6-month follow-up (P=.01). Similarly, Sengupta et al [84] reported that midlife women showed statistically significant improvement in waist circumference (P=.048), weight (P=.02), and BMI (P=.01) from baseline.

#### Sleep Disturbance

The impact of a mobile-based PA intervention (Garmin Vivofit 2 wearable and its paired app) on sleep quality measured by ActiGraph and the Pittsburgh Sleep Quality Index was reported as a secondary analysis of the Activity and Technology (ACTIVATE) RCT on menopausal survivors of breast cancer [83]. At 12 weeks of intervention, a significant reduction in both actigraphy-based awake time after sleep and number of awakenings equivalent to −5.7 minutes (95% CI −11.7 to −0.2) and −2.0 minutes (95% CI −3.6 to −0.4) was observed, respectively, compared with the control arm. The changes in Pittsburgh Sleep Quality Index scores and actigraphy sleep efficiency favored the intervention arm, although there was no statistically significant difference between the groups [83].

#### QoL Measure

The study by Kashifi et al [76] measured the impact of a mobile-based PA intervention using WhatsApp on the QoL of menopausal women in Iran using the self-reported Menopause-Specific Quality of Life Questionnaire at baseline and the 1-month intervention follow-up. The study showed significant improvements in vasomotor, physical, and psychosocial dimensions between the intervention and control groups 1 month after the intervention. The mean difference in total QoL between the 2 groups was −10.52 (P<.001). Within the intervention group, the total QoL dimension changed significantly from 72.70 (SD 5.33) at baseline to 63.81 (SD 6.81), with lower scores indicating better QoL [76].

#### Psychosocial Outcomes

The impact on perceived stress and depressive symptoms was assessed in 8% (1/12) of the studies [84] using the adapted rating scores of the Perceived Stress Scale and the Patient Health Questionnaire-9. Sengupta et al [84] showed nonsignificant improvements in perceived stress scores, possibly because of the limited functionality of the prototype, with a significant reduction in depressive symptoms observed by the end of the 12-week intervention.

### Experienced Acceptability: Quantitative Data

The usability and acceptance of the interventions were examined quantitatively in 42% (5/12) of the studies using surveys [56,78,79,81,84] (Table 7). Acceptability was most frequently assessed in terms of satisfaction and the users’ experience of using mobile apps, WATs, or the overall program. There were high levels of satisfaction and acceptability, favoring the use of mobile apps and Fitbit activity trackers. For instance, Cadmus-Bertram et al [78] compared a Fitbit-based intervention with a traditional pedometer without feedback received in a control group and found significantly higher satisfaction levels in the Fitbit group—96% (24/25) rated Fitbit as “somewhat or very helpful” compared with only 32% (8/26) in the pedometer control group. Similarly, 67% (2/3) of the pre-post studies [56,79] found that midlife women favored Fitbit (combined use of the Fitbit app and activity tracker) and reported that using Fitbit was “motivational to PA.”

**Table 7:** Acceptability ratings across the included quantitative studies.

Author, year, intervention type	Acceptability measurements informed by the TAM2^a^ model [72]	Acceptability rating
Cadmus-Bertram et al [78], 2016, Fitbit-based intervention (activity tracker and app-based website)	User experience and satisfaction survey Perceived ease of use Perceived usefulness Intention and likelihood of future use	A total of 96% (24/25) of midlife women liked the Fitbit app-based website. There were lower perceived barriers associated with the use of Fitbit; 80% (20/25) reported no technical issues or difficulty with the trackers. A total of 96% (24/25) rated Fitbit as “somewhat or very helpful” for increasing PA^b^ compared with only 32% in the pedometer control group. A total of 76% (19/25) reported that they would recommend Fitbit to a friend.
Valle et al [81], 2017, weight loss mobile intervention (tracker and app)	Program acceptability and satisfaction survey	Almost all the intervention group (11/11) was satisfied and rated the tracker as “extremely helpful” on a 4-point scale at 6 months.
Butryn et al [79], 2016, Fitbit-based intervention (tracker and app)	Satisfaction survey Perceived confidence Intention and likelihood of future use	At 6 months, 89% (25/28) of the participants rated the whole program as favorable for increasing PA on a 5-point Likert scale (mean 4.11 out of 5, SD 1.14). The Fitbit was reported as the “best part.” After the intervention ended, 88% (24/28) reported confidence in the ability to maintain PA over the next 3 months. At 6 months, 77% (22/28) reported that they had purchased or intended to purchase a device. In total, 88% (24/28) agreed to recommend the program to others.
Sengupta et al [84], 2020, HerBeat mobile app and smartwatch	User satisfaction using the SUS^c^ Perceived usefulness and ease of use	Midlife women found the app features to be easy to use and well integrated (mean score on the SUS was 83.60, SD 16.4). Participants somehow felt confident in using the app. The most frequent technical complaints were regarding the short battery life of the smartwatch. Participants had no adverse events or privacy concerns.
Joseph et al [56], 2021, Fitbit-based intervention (tracker and app)	Consumer satisfaction survey	Treatment acceptance was measured using an adapted consumer satisfaction survey to assess users’ perceptions of the intervention’s content, app usability, and preferences. A total of 87% (13/15) of the women found the combined use of the Fitbit app and activity tracker helpful and “motivational to exercise.”

^a^TAM2: technology acceptance model 2.

^b^PA: physical activity.

^c^SUS: System Usability Scale.

The perceived usefulness and ease of use, where measured, were often limited to whether users experienced technical issues associated with the use of mobile apps and activity trackers. Cadmus-Bertram et al [78] found that 80% (20/25) of midlife women had no technical difficulties with the Fitbit trackers and reported technical issues that were easy to resolve. Furthermore, participants reported that more hands-on training could improve their satisfaction and engagement with the app-based website functions. Sengupta et al [84] reported on the acceptability and usability of the HerBeat smartwatch and paired app. In this pilot study, midlife women with cardiovascular diseases found the app features to be easy to use but complained about the short battery life of the HerBeat smartwatch [84].

Across the included studies, no adverse events were reported by the participants themselves or by the research team to be related to the use of mobile apps or trackers.

### Anticipated Acceptability: Qualitative Data

We identified three main themes from 33% (4/12) of high-quality studies related to perceived usefulness, readiness to use, and ease of using mobile PA technologies [75,77,79,82]. A summary description of the themes with some corresponding excerpts can be found in Multimedia Appendix 4 [75,77,79,82].

#### Theme 1: Perceived Usefulness to Increase Self-awareness of PA and Menopause Experience

Mobile apps were viewed as an opportunity to track self-management behaviors such as exercise, dietary intake, and regular health checkups that could support the management of menopausal symptoms [75,77,79,82]. Promoting and tracking PA during menopause was perceived as a critical feature of a mobile-based intervention to increase self-awareness of PA and sedentary time, particularly in working women who were less aware of their sitting time [80].

Similarly, Lee et al [75] reported the need for an app to encourage exercise and contain personalized health management information as most participants wanted self-management strategies to facilitate lifestyle changes other than receiving medical treatments, as well as a space where menopausal women could share common experiences.

#### Theme 2: Perceived Readiness and Ease of Using Mobile Apps and Activity Trackers

Midlife women appeared to have some level of hesitancy and lack of readiness to adopt and engage with mobile PA technologies that were rooted in their perceived low confidence with technology and limited knowledge and technological capabilities regarding how to use the devices and in the complexity of WATs that could intimidate midlife women into ending up not using the technologies [77,80].

There were mixed views among midlife women on perceived ease of use of WATs and their paired apps. Most midlife women from the focus group by Nguyen et al [80] had no trouble using commercially available trackers and their apps (eg, *Garmin Vivofit 2*, *Fitbit*, and *Polar A300*), yet most of them relied on basic features of activity trackers, such as the step-counting function. By contrast, some women had limited use of functions as they found it challenging to set up wearables and synchronize them with their phones [80]. Hands-on training could support midlife women in setting up and ensuring ease of use of mobile PA technologies. Simplicity of content, clear communication and navigation, and appropriate use of colors and text were considered important to ensure user-friendliness [80,82]. A participant highlighted the importance of ensuring that positive language is used when referring to menopause to empower midlife women through their menopause journey [77].

However, midlife women experienced challenges associated with the practicality of activity trackers that discouraged their motivation and intention to wear and sustain the use of PA trackers over time. These challenges included discomfort of wearables, particularly regarding size or buzzing; inability to record light-intensity PA and strength training; and concerns about accuracy. Subsequently, some participants reported disuse of wearables over time as they were not enough to maintain PA or reported that they ignored alarms because of frustration [80].

Midlife women also emphasized the significant value of the esthetics of wearables to determine their preferences and likelihood of using PA trackers. Midlife women preferred smaller activity trackers such as *Fitbit* and *Garmin* Vivofit 2 [80]. However, some participants reported that trackers with larger screens and text would be easier to see and push buttons in [80].

#### Theme 3: Midlife Women’s Favored Features of PA Apps

##### Step Count

Step counting was the most favored feature of mobile apps and activity trackers, with little use of other advanced features of mobile apps or WATs [80]. Menopausal women found that calculating and viewing the number of steps was helpful in hitting 10,000 steps a day.

##### Setting Goals and Monitoring Progress

Midlife women expressed their desire to use a PA app that allowed for goal setting and daily step-count monitoring to guide behavior changes to eventually help minimize burdensome menopausal symptoms in their busy lives [77,80,84] as this was seen as motivational [80]. However, most participants found that a feature that automatically adjusted the user’s step goal based on previous activity levels was less motivational compared with fixed or manually adjusted goals [80].

##### Real-time Feedback of PA

Receiving notifications on smartphones to encourage PA was perceived as acceptable to nudge women to exercise. Apps with personalized notifications were felt to be more effective based on how motivational and nonrepetitive the prompts were [75]. Midlife women also liked the idea of receiving real-time feedback on PA behavior on the apps. Some participants also acknowledged the additional positive reinforcement via emails, SMS text messages, or peer support via social media sites [80].

### BCT Identification

**Figure 3:**
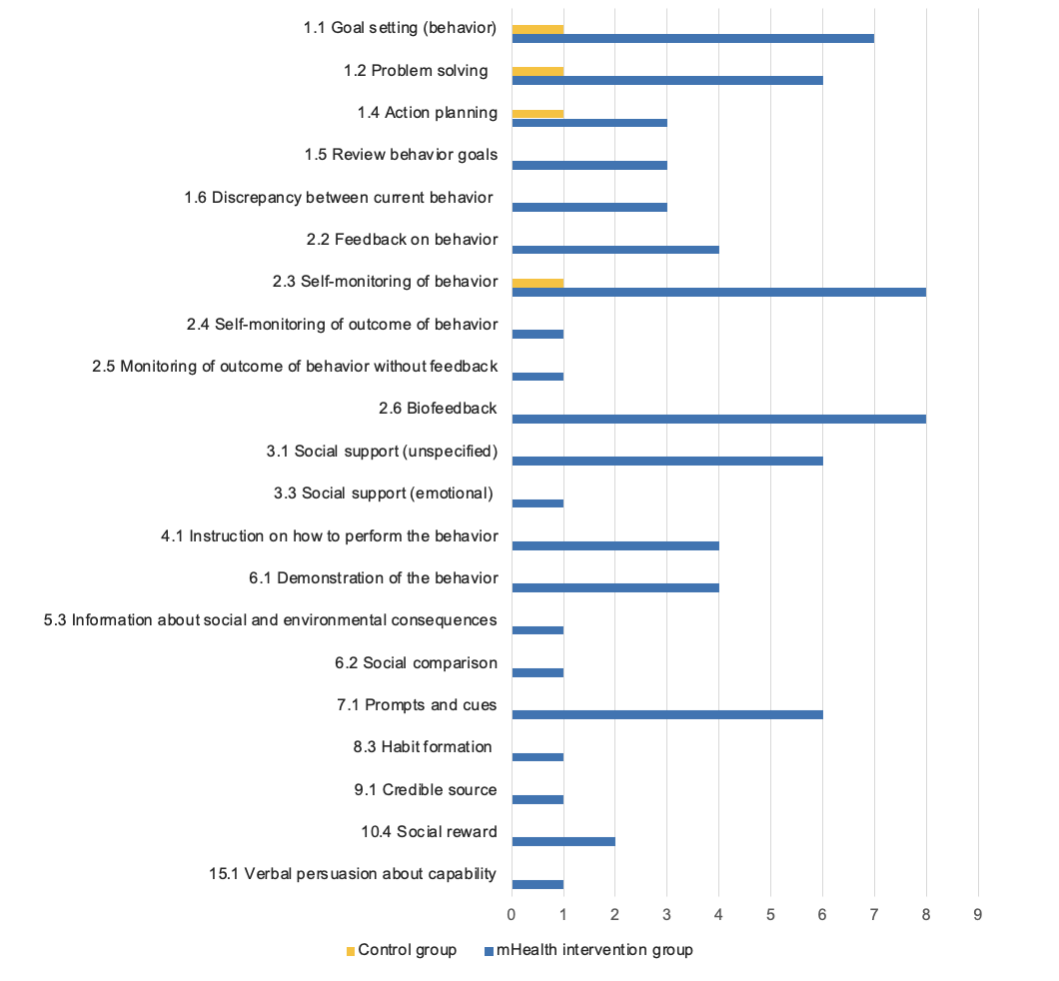
The frequency of coded behavior change techniques (BCTs) across the included interventions (n=9). mHealth: mobile health; PA: physical activity.

The average number of BCTs per mobile PA intervention was 8.8 (range 4-13; Figure 3). BCTs were mapped out for all actual interventions included (9/12, 75%) and the one active control (1/12, 8%) in the study by Cadmus-Bertram et al [78] (Table 6).

Collectively, 22 different BCTs from 9 different clusters of the BCTTv1 were identified across the 75% (9/12) of coded interventions. A total of 8% (7/93) of the BCTs were used in more than half of the interventions—“Self-monitoring of behaviour” (8/9, 89%), “Biofeedback” (8/9, 89%), and “Goal setting (behaviour)” (7/9, 78%) were the most frequently described BCTs. “Problem solving,” “Social support (unspecified),” “Prompts and cues,” and “Information on health consequences” were identified in 67% (6/9) of the interventions each. All the included interventions (9/9, 100%) used at least one BCT from cluster 1 “Goals and planning” or cluster 2 “Feedback and monitoring” of the BCTTv1.

There were no clear patterns between the type and total number of BCTs used and the effectiveness of the included interventions. In total, 50% (2/4) of the RCTs [76,83] reported significant evidence on PA, sedentary time, sleep disturbance, and QoL outcomes and used a total of 10 and 8 BCTs. By contrast, the RCTs by Cadmus-Bertram et al [78] and Valle et al [81] used 6 and 12 BCTs, respectively, yet found no evidence of effectiveness. In the RCT by Cadmus-Bertram et al [78], the distinction in BCTs between the intervention (Fitbit-based) and active control (pedometer-based) groups was feedback on behavior, biofeedback, and review behavior goals, which were only present in the intervention group. Both groups involved the common BCTs “Goal setting (behaviour),” “Action planning,” and “Self-monitoring of behaviour” [78].

In total, 67% (2/3) of the pre-post studies [56,79] found some supporting evidence of significant changes in PA, sedentary time, and weight loss from baseline and used 10 and 8 BCTs. However, Sengupta et al [84] used 9 BCTs and reported no evidence of the effectiveness of mobile apps on PA, perceived stress, and depressive symptoms (see Multimedia Appendix 5 [56,76,78-81,83-85] for the full coding process of BCTs of each intervention studied).

#### Theoretical Consideration

A total of 42% (5/12) of the studies mentioned the contribution of behavioral theories to inform the design and development of the mobile PA technology. Four specific theories were referenced: the Coventry, Aberdeen, and London-Refined (CALOR-E) [78]; Behavior Change Support Systems [82]; self-regulation theory [81]; and SCT [56,85]. Of these 5 theory-informed studies, 2 (40%) were qualitative and had no evaluation outcomes [82,85]. In total, 40% (2/5) of comparator studies reported no significant evidence on both PA and weight outcomes. Only the trial by Joseph et al [56] was based on SCT and found some supporting evidence of a significant increase in PA of 30 minutes per week over 4 weeks (P<.001). The reporting of theoretical underpinnings in the development and implementation of interventions was generally poor.

## Discussion

### Principal Findings

This review of 12 studies found that mobile PA interventions in the form of stand-alone apps or WATs resulted in a small to moderate increase in objectively measured MVPA of approximately 61.36 minutes per week among midlife women, at least in the short term (≤16 weeks). However, precision decreases with a reduced sample size and, thus, the pooled effect size should be interpreted with caution. Although a meta-analysis was not possible for other menopause-related outcomes, moderate- to high-risk evidence suggests significant, positive effects on weight reduction, managing anxiety and sleep disturbance, and enhancing menopause-specific QoL domains in midlife women. Quantitative studies were mostly uncontrolled with small sample sizes. We also found from high-quality qualitative exploratory research that most midlife women perceived mobile technologies as acceptable and potentially helpful in motivating them to increase PA levels. Daily step count was seen as an acceptable and clear outcome to monitor.

To our knowledge, this is the first systematic review to synthesize the current evidence with regard to the effectiveness and acceptability of mobile apps and WATs targeting PA in midlife women. An increase of 61.36 minutes per week, at least in the short term, among both healthy and clinical midlife women is promising. PA apps and WATs tend to be effective in comparison with no intervention or traditional pedometer-based interventions with no mobile technology component. The estimated increase in weekly MVPA represents 40.9% (61/150) of the recommended weekly MVPA for adults aged 19 to 64 years [89]. However, certainty in the pooled effect estimate was downgraded because of small study bias. Although all other individual effect estimates favored mobile PA interventions, not all studies reached statistical significance.

This review was consistent with the recently published meta-analysis of 63 studies (N=8250 participants) of digital and mechanical wearables providing PA feedback showing a small pooled effect for MVPA equivalent to 48.5 minutes per week (95% CI 33.8-63.3) among adult populations [90]. Similarly, the meta-analysis by Laranjo et al [91] found that mobile apps and WATs caused small to moderate increases in PA (equivalent to 1850 steps per day) among healthy adult populations. The meta-analysis by Yerrakalva et al [92] also found a modest increase of 753 steps per day among older adults after using app-based interventions for ≤3 months. Owing to substantial physical inactivity among midlife women compared with the general adult population [5,7], even small increases in MVPA are likely to be beneficial. The effects of mobile PA interventions on sedentary behavior in this population as sedentary time were inconclusive, highlighting a need for more research to assess the impact of mobile apps on sedentary behavior outcomes in midlife women [79,83].

Few studies (6/12, 50%) evaluated menopause-related outcomes. We found mixed evidence of the effect on weight loss [78,79,81]. Single studies found positive effects on sleep disturbance [83], menopause-specific QoL [76], and depressive symptoms but not on perceived stress [84]. None of the included studies assessed the effect of mobile PA interventions on vasomotor symptoms such as hot flushes and night sweats.

### Design of Acceptable, Potentially Effective Mobile PA Interventions for Midlife Women

In alignment with the key constructs of the TAM [72], findings from the qualitative synthesis suggest that perceived usefulness was grounded in women’s beliefs about the extent to which mobile apps or WATs could increase self-awareness of PA and improve the overall menopause experience by exchanging reliable health information and promoting behavior change. There was a tendency to favor a holistic approach when designing apps for midlife women by focusing on menopause as a whole experience and on lifestyle behaviors rather than on the limited functions of menopausal symptom trackers [77].

In this review, midlife women showed a desire for PA mobile technology that required only limited technical abilities and met their needs and preferences without imposing further burdens on their busy lives [77,80,82]. Echoing previous studies in older populations [93-96], women aged 40 to 64 years showed some hesitancy toward new technology and tended to be reluctant to take up mobile apps and WATs, possibly because of low confidence and SE in using new technology [82]. This may, in turn, reduce the effect of trackers on PA behavior change [97]. However, unlike a common reluctance to learn new technology among older populations [98,99], our review findings suggest that midlife women were willing to learn how to use apps to increase PA and make better lifestyle changes [77].

One of the key findings is related to which specific features of mobile apps and WATs were preferred by midlife women. Previous research suggests that effective PA apps for the general population might need some adaptations to meet the needs and requirements of each subgroup of that population [100]. The qualitative synthesis suggests that midlife women liked simple features, namely, step goal setting, activity monitoring, real-time feedback, easy-to-read content, and a user-friendly interface, with most midlife women considering step counting as the most favored feature [82,83]. We noticed that midlife women shared similar preferences related to functionality with older populations [95,99,101,102]. For instance, using large visual screens and readable text was perceived as helpful [91,93] and, thus, may facilitate PA mobile technology use among midlife women.

Furthermore, the application of the TAM suggests that providing technical support may facilitate uptake and engagement with new technologies [72]. Access to additional telephone or face-to-face technical support may increase midlife women’s confidence in technology, especially among those who were initially reluctant [78,80]. Hands-on training and easy-to-read manuals to guide the installation, synchronization of PA apps, and use of WATs were perceived as essential.

### Most Frequently Reported BCTs

“Self-monitoring,” “biofeedback,” and “goal setting of PA behaviour” were the most frequently used BCTs across the included studies. These findings concur with previous literature on digital behavior change interventions targeting PA, which also highlighted the role of “social support” in adults [103-105] and older adults [106]. In this review, “social support” was used in 67% (6/9) of the studies targeting both healthy [56,76,79,85] and clinical (ie, survivors of breast cancer) [81,83] midlife women. Most recently, research suggests that midlife women are ready to make positive behavior changes, yet they need social support and connectivity [107,108]. Similarly, our qualitative synthesis found that midlife women would prefer an app that offers a safe space to share common experiences and receive social support [75].

However, because of the scarcity of existing evidence and heterogeneity in intervention type (eg, smartphone apps and WATs), multicomponent interventions (eg, in-person sessions, SMS text messaging, and follow-up calls), mode of delivery, and outcomes measured, it was not possible to ascertain which intervention components or BCTs were most effective in increasing PA or improving menopause-related outcomes. Reporting of interventions and mode of delivery in the included studies was insufficient; accordingly, we could not comment on the link between the described BCTs and mechanisms of action, a problem highlighted in similar reviews. Sediva et al [103] highlighted the relevant real concern of low treatment fidelity on the delivery of content as planned across the 13 included complex interventions, including PA apps. By contrast, without adequate information reported on measurements of fidelity or ensuring that the underpinning theory is reflected in the design and implementation process, implementation failure can potentially occur and, thus, the real-world effectiveness of such interventions must be considered with caution [109,110].

Interestingly, the top identified BCTs—“self-monitoring,” “goal setting,” and “biofeedback”—were in parallel with the most preferred app features perceived by midlife women according to the qualitative data synthesis. Hence, to optimize the effectiveness of mobile PA interventions in midlife women, it might be beneficial for future mobile interventions to take advantage of the simple features of step counts, goal setting, and real-time feedback and pair them with a sufficient number of BCTs. Evidence suggests that incorporating more BCTs is more effective than using limited or single BCTs to obtain significant effects on PA [106,111]. Further research is needed to determine which mobile PA components or active BCTs are the most effective in increasing PA in midlife women.

### Strengths, Limitations, and Future Implications

To our knowledge, this is the first systematic review evaluating the use of mobile PA interventions among midlife menopausal women. The main strengths of this review are the rigorous and inclusive methodological approach and the comprehensive and extensive literature search. The screening, data extraction, and risk-of-bias assessment processes were independently reviewed by a second researcher. BCT coding was also independently conducted by 2 trained researchers, with high agreement.

However, this review has certain limitations. It should be noted that the findings of this review were based on healthy and clinical midlife women with potentially chronic conditions as well as on survivors of breast cancer, which may reflect special needs and, thus, different perspectives toward using mobile apps and WATs. For example, Nguyen et al [80] highlighted that survivors of breast cancer may have higher motivation to exercise to prevent recurrence of cancer and, thus, to sustain the use of WATs than healthy women. In this review, we only identified 25% (1/4) of RCTs [78] that targeted middle-aged women from the general population. However, the results were relatively consistent across populations. Future research should focus on targeting midlife women from the general community to ensure the generalizability of the findings.

In total, 58% (7/12) of the studies were at moderate to high risk of bias, and the inclusion of lower-quality study designs (eg, pre-post studies) substantially increased the risk of bias. Moreover, the data extracted from most of the included quantitative studies did not adjust for covariates and, thus, the meta-analysis reflects an unadjusted effect. Owing to the inherent variability in mobile interventions, control groups (active or no intervention), and the methodological quality of the included studies, the effect sizes of the pooled estimate and from individual studies had large CIs and low precision. Hence, further studies with larger sample sizes are needed to measure outcomes in a consistent way as the current lack of significant results of individual studies could be attributed to either a lack of effect or underpowered studies.

Given the novelty of this research area and the scarcity of existing RCTs, the inclusion of different study designs provides valuable insights. A total of 25% (3/12) of the studies included in this review were exploratory, participatory design qualitative studies that involved midlife women to obtain their insights before developing innovative mobile solutions, which may enhance the relevance and uptake of such interventions by this population. However, further rigorous studies informed by relevant theoretical frameworks and best practices are essential to explore how midlife women and their subgroups can best participate in the design of mobile PA interventions. Furthermore, qualitative process evaluations of interventional studies might also be equipped to fill in the gaps regarding the experiences and engagement with mobile PA interventions among midlife women.

Few empirical studies looked at the effect of PA mobile technologies [19,20,23] on menopause-related outcomes. Menopause is a life transition affecting half of the population and, thus, an area requiring further research and innovative applications. Rigorous studies on menopause-related outcomes can then serve as a solid base for policies and interventions to support more inclusive workplaces for women. For instance, promoting mobile apps for PA may be a way that workplaces and health care settings could use as a scalable, cost-effective strategy to deal with menopausal symptoms.

### Conclusions

The findings from this review suggest that mobile PA interventions in the form of apps and WATs are likely to be acceptable to midlife women and may potentially increase PA. Evidence was mixed for sedentary time and weight loss, with single studies suggesting positive improvements in sleep disturbance and menopause-specific QoL domains. The most frequently reported BCTs across the included studies were biofeedback, self-monitoring of behavior, and goal setting (behavior). The most acceptable components of PA apps were manual goal setting and step trackers, whereas activity trackers needed to be comfortable and attractive. Although the approach of using mobile PA apps in midlife women appears promising, larger, high-quality studies should address the lack of evidence on effectiveness, acceptability, and feasibility of using mobile PA apps to address menopause-related outcomes and, thus, encourage midlife women to seek support to manage menopausal symptoms.

## Appendix

Multimedia Appendix 1 Search strategy.

Multimedia Appendix 2 Reasons for study exclusion.

Multimedia Appendix 3 Interrater reliability between independent reviewers.

Multimedia Appendix 4 Thematic analysis of qualitative studies and excerpts.

Multimedia Appendix 5 Behavior change technique coding process.

Multimedia Appendix 6 PRISMA (Preferred Reporting Items for Systematic Reviews and Meta-Analyses) checklist.

## Abbreviations

ACTIVATE: Activity and Technology
BCT: behavior change technique
BCTTv1: Behavior Change Technique Taxonomy v1
CALOR-E: Coventry, Aberdeen, and London-Refined
IPAQ-SF: International Physical Activity Questionnaire-Short Form
MVPA: moderate to vigorous physical activity
PA: physical activity
PPAQ: Paffenbarger Physical Activity Questionnaire
PRISMA: Preferred Reporting Items for Systematic Reviews and Meta-Analyses
QoL: quality of life
RCT: randomized controlled trial
SCT: Social Cognitive Theory
SE: self-efficacy
TAM: technology acceptance model
WAT: wearable activity tracker

## Data Availability

Data extraction and quality assessment templates can be available upon request.
