# Supplementary material for: Assessing the Acceptability and Effectiveness of Mobile-Based Physical Activity Interventions for Midlife Women During Menopause: Systematic Review of the Literature
Source: JMIR Mhealth Uhealth. 2022 Dec 9;10(12):e40271. doi: 10.2196/40271 (PMC9789501; doi:10.2196/40271)
Supplement: Multimedia Appendix 1 [file mhealth_v10i12e40271_app1.docx]

**Multimedia Appendix 1: Search Strategy**

Database: Ovid MEDLINE(R) ALL <1946 to June 13, 2022>

Search Strategy:

--------------------------------------------------------------------------------

1 exp Climacteric/ or Climacteric.mp. (65538)

2 exp Menopause/ (61340)

3 Menopaus* (tw,kf) (54439)

4 exp Postmenopause/ (26868)

5 (Postmenopaus* or Post-menopaus*) (tw,kf) (66619)

6 exp Perimenopause/ (1521)

7 (peri-menopaus* or perimenopaus*) (tw,kf) (5387)

8 exp Hot Flashes/ (3495)

9 (hot-flash* or hot flash* or hot-flush* or hot flush*) (tw,kf) (5040)

10 night sweat* (tw,kf) (2693)

11 nocturnal sweat* (tw,kf) (108)

12 (vasomotor adj2 symptom*) (tw,kf) (2441)

13 or/1-12 (121256)

14 (mHealth or m-Health) (tw,kf) (8361)

15 (Smartphone* adj3 application*) (tw,kf) (3761)

16 (mobile adj3 application*) (tw,kf) (7136)

17 (mobile and (app or apps or application*)) (tw,kf) (25749)

18 (Smartphone* and (app or apps or application*)) (tw,kf) (10397)

19 (mobile adj3 intervention*) (tw,kf) (2352)

20 Medical Informatics Applications.mp. or exp Medical Informatics Applications/ (478018)

21 exp Telemedicine/ (40738)

22 Telemedicine (tw,kf) (20804)

23 (telehealth or tele-health) (tw,kf) (10778)

24 (ehealth or e-health) (tw,kf) (9413)

25 Wearable* (tw,kf) (21057)

26 (Self-monitor* or Self monitor*) (tw,kf) (9548)

27 (Mobile adj2 tracker*) (tw,kf) (119)

28 (fitness app or fitness apps or fitness application*) (tw,kf) (144)

29 exp Fitness Trackers/ (1020)

30 Activity tracker* (tw,kf) (859)

31 Step* counter (tw,kf) (103)

32 exp Cell Phone/ or exp Smartphone/ (20485)

33 activity monitor* (tw,kf) (4418)

34 fitbit* (tw,kf) (994)

35 (armband* or arm band*) (tw,kf) (752)

36 fitness watch* (tw,kf) (7)

37 pedomet* (tw,kf) (3037)

38 accelerometer* (tw,kf) (17487)

39 fitness monitor* (tw,kf) (64)

40 (smartwatch* or smart watch*) (tw,kf) (900)

41 sports watch* (tw,kf) (26)

42 (wristband* or wrist band*) (tw,kf) (726)

43 (tablet* and (mobile or computer or based intervention)) (tw,kf) (4881)

44 iphone* (tw,kf) (1114)

45 ipad* (tw,kf) (1822)

46 (android* and (mobile or app or play store)) (tw,kf) (1558)

47 Itunes (tw,kf) (233)

48 mobile technolog* (tw,kf) (2759)

49 (digital adj2 intervention*) (tw,kf) (1877)

50 (Sweatcoin or MyFitnessPal or Freeletics or Strava or Zombies or Nike+Run Club or Fitness Buddy or MayMyRun or RunKeeper or Runtastics) (tw,kf) (197)

51 (IOS and (mobile or app or apps)) (tw,kf) (697)

52 or/14-51 (623217)

53 exp Exercise Therapy/ (59887)

54 exp Physical Exertion/ or exp Physical Fitness/ (89502)

55 exp Exercise/ (232655)

56 physical activit* (tw,kf) (138675)

57 physical training* (tw,kf) (6427)

58 exercis* (tw,kf) (341178)

59 fitness (tw,kf) (89179)

60 exp Sports/ (206747)

61 exp Muscle Stretching Exercises/ or muscle stretching (tw,kf) (2321)

62 athletic (tw,kf) (18268)

63 exp Yoga/ or yoga (tw,kf) (6473)

64 (tai chi or Qi Gong) (tw,kf) (2210)

65 exp Walking/ or walk* (tw,kf) (160799)

66 treadmill (tw,kf) (35069)

67 exp Jogging/ or exp Running/ or jog* (tw,kf) (24641)

68 exp Swimming/ or Swim* (tw,kf) (56308)

69 aerobic* (tw,kf) (99858)

70 Pilate* (tw,kf) (719)

71 exp Movement/ or movement (tw,kf) (834679)

72 cycling (tw,kf) (72777)

73 (inactive or inactivity or gym or gyms) (tw,kf) (119084)

74 or/53-73 (1591887)

75 13 and 52 and 74 (245)
