# Supplementary material for: Assessing the Acceptability and Effectiveness of Mobile-Based Physical Activity Interventions for Midlife Women During Menopause: Systematic Review of the Literature
Source: JMIR Mhealth Uhealth. 2022 Dec 9;10(12):e40271. doi: 10.2196/40271 (PMC9789501; doi:10.2196/40271)
Supplement: Multimedia Appendix 2 [file mhealth_v10i12e40271_app2.docx]

**Multimedia Appendix 2: Reasons for Study Exclusion**

| Excluded Studies | Reason for exclusion |
| --- | --- |
| Abedi P, Nikkhah P, Najar S. Effect of pedometer-based walking on depression, anxiety and insomnia among postmenopausal women. Climacteric. 2015;18(6):841-5. doi: 10.3109/13697137.2015.1065246. Epub 2015 Sep 23. PMID: 26100101. | Intervention: Pedometer-based intervention |
| Abedi P, Nikkhah P, Najar S. Effect of pedometer-based walking on depression, anxiety and insomnia among postmenopausal women. Climacteric. 2015;18(6):841-5. doi: 10.3109/13697137.2015.1065246. Epub 2015 Sep 23. PMID: 26100101 | Duplicate publication |
| Pudkasam S, Pitcher M, Fisher M, O'Connor A, Chinlumprasert N, Stojanovska L, Polman R, Apostolopoulos V. The PAPHIO study protocol: a randomised controlled trial with a 2 x 2 crossover design of physical activity adherence, psychological health and immunological outcomes in breast cancer survivors. BMC Public Health. 2020 May 15;20(1):696. doi: 10.1186/s12889-020-08827-x. PMID: 32414347; PMCID: PMC7227193. | Publication: Protocol Participant: Not targeting women at menopause age / Outcome: Cancer-specific outcome measurements |
| Scott. Intelligent Personal Assistants for Delivering Telehealth to Postmenopausal Women with Osteoporosis (2021) | Protocol/ Digital intervention identified (VIPA) doesn't target PA behaviour change/ irrelevant outcome |
| Pudkasam et al . Kowhai Study: The Younger Women's Wellness Program After Breast Cancer - A feasibility study of an e-health enabled lifestyle modification intervention ACTRN12620000260921. 2020(10) <https://trialsearch.who.int/Trial2.aspx?TrialID=ACTRN12620000260921> | Publication: Protocol / Intervention: doesn't fall under the working definition of PA mHealth/ Cancer-specific outcome measurements / Participant: Adults age breast cancer survivors, Not targeting women at menopause age |
| Alonso-Domínguez R, Sánchez-Aguadero N, Llamas-Ramos I, Lugones-Sánchez C, González-Sánchez S, Gómez-Marcos MA, García-Ortiz L; Ewomen Investigators. Effect of an intensive intervention on the increase of physical activity and the decrease of sedentary lifestyle in inactive postmenopausal. J Adv Nurs. 2021 Apr;77(4):2064-2072. doi: 10.1111/jan.14737. Epub 2021 Jan 22. PMID: 33481300. | Publication: Protocol |
| Anderson AS, Chong HY, Craigie AM, Donnan PT, Gallant S, Hickman A, McAdam C, McKell J, McNamee P, Macaskill EJ, Mutrie N, O'Carroll RE, Rauchhaus P, Sattar N, Stead M, Treweek S. A novel approach to increasing community capacity for weight management a volunteer-delivered programme (ActWELL) initiated within breast screening clinics: a randomised controlled trial. Int J Behav Nutr Phys Act. 2021 Mar 6;18(1):34. doi: 10.1186/s12966-021-01099-7. Erratum in: Int J Behav Nutr Phys Act. 2022 Jan 11;19(1):4. PMID: 33676538; PMCID: PMC7936444. | Intervention: Not considered as mobile digital intervention (pedometer-based walking) |
| Anderson, D., Seib, C., Tjondronegoro, D. et al. The Women’s wellness after cancer program: a multisite, single-blinded, randomised controlled trial protocol. BMC Cancer 17, 98 (2017). https://doi.org/10.1186/s12885-017-3088-9 | Intervention: Telehealth which doesn't fall under consumer-facing PA mHealth applications or wearables |
| M. Tadayon, P. Abedi and F. Farshadbakht (2015). Impact of pedometer-based walking on menopausal women's sleep quality: a randomized controlled trial. Climacteric. <http://dx.doi.org/10.3109/13697137.2015.1123240> | Duplicate publication |
| Arigo, et al (2017). FEASIBILITY OF A FITBIT-SUPPORTED BEHAVIORAL INTERVENTION TO INCREASE PHYSICAL ACTIVITY AMONG OBESE POST-MENOPAUSAL WOMEN | Publication: Symposium, No full-text paper |
| Befort CA, Klemp JR, Austin HL, Perri MG, Schmitz KH, Sullivan DK, Fabian CJ. Outcomes of a weight loss intervention among rural breast cancer survivors. Breast Cancer Res Treat. 2012 Apr;132(2):631-9. doi: 10.1007/s10549-011-1922-3. Epub 2011 Dec 25. PMID: 22198470; PMCID: PMC3314288. | Intervention: conference calls (telehealth) not falling under the definition of mHealth |
| Bellefeuille P, Robillard ME, Ringuet ME, Aubertin-Leheudre M, Karelis AD. Comparison between several muscle strength and cardiorespiratory fitness indices with body composition and energy expenditure in obese postmenopausal women. Int J Sports Med. 2013 Mar;34(3):258-62. doi: 10.1055/s-0032-1321802. Epub 2012 Sep 12. PMID: 22972252. | Study Design: Cross-sectional (correlation study) with no digital intervention to increase PA |
| Bogucka A, Kopiczko A, Glebocka A. The effects of selected lifestyle components on the risk of developing dynapenia in women – a pilot study. Anthropological Review; Poznan[Vol. 81, Iss. 3,](https://www.proquest.com/indexingvolumeissuelinkhandler/1976405/Anthropological+Review/02018Y01Y01$232018$3b++Vol.+81+$283$29/81/3;jsessionid=203A7CF6F9FE35AE920319D86A9428EA.i-0fbbf2c7a81cd5373) (2018): 289-297. DOI:10.2478/anre-2018-0023 | Participants: +65. Irrelevant outcome, Intervention: No digital intervention used. Pedometer was used to measure PA |
| Borrie, et al. (2018). Jazzexercise supervisied program | Intervention: Jazzexercise supervisied program with no mobile digital intervention/  Publication: Conference Proceedings / No PA intervention (control). |
| Cadmus-Bertram L, Marcus BH, Patterson RE, Parker BA, Morey BL. Use of the Fitbit to Measure Adherence to a Physical Activity Intervention Among Overweight or Obese, Postmenopausal Women: Self-Monitoring Trajectory During 16 Weeks. JMIR Mhealth Uhealth. 2015 Nov 19;3(4):e96. doi: 10.2196/mhealth.4229. PMID: 26586418; PMCID: PMC4705008. | Duplicate intervention of included RCT |
| Cadmus-Bertram, et al (2015). USE OF THE FITBIT TO MEASURE ADHERENCE TO A PHYSICAL ACTIVITY INTERVENTION IN POSTMENOPAUSALWOMEN | Duplicate publication |
| Cadmus-Bertram L, Wang JB, Patterson RE, Newman VA, Parker BA, Pierce JP. Web-based self-monitoring for weight loss among overweight/obese women at increased risk for breast cancer: the HELP pilot study. Psychooncology. 2013 Aug;22(8):1821-8. doi: 10.1002/pon.3219. Epub 2012 Nov 27. PMID: 23188655. | Intervention: counselling calls and web-based intervention + pedometer, doesn't fall under mHealth emrging applications. No report of menopausal symptoms |
| Cochrane Central Register of Controlled Trials (CENTRAL) (2016). Effects of exercise training with power control in muscle strength, cholesterol and body fat postmenopausal women. RBR-9cbp8s CN-01827424 31. 2019(3). <https://trialsearch.who.int/Trial2.aspx?TrialID=RBR-9cbp8s> | Intervention: No digital mobile intervention applied |
| Chihara H, Kawase R, Otsubo Y, Hiraizumi Y, Takeshita T. Effect of insulin resistance improvement due to lifestyle intervention on overweight perimenopausal Japanese women: a preliminary study. J Nippon Med Sch. 2008 Feb;75(1):15-22. doi: 10.1272/jnms.75.15. PMID: 18360074. | Intervention: No digital mobile intervention applied (Accelerometer was used as outcome objective measure) |
| Coll-Risco I, Acosta-Manzano P, Borges-Cosic M, Camiletti-Moiron D, Aranda P, Soriano-Maldonado A, Aparicio VA. Body Composition Changes Following a Concurrent Exercise Intervention in Perimenopausal Women: The FLAMENCO Project Randomized Controlled Trial. J Clin Med. 2019 Oct 14;8(10):1678. doi: 10.3390/jcm8101678. PMID: 31615008; PMCID: PMC6832269. | Intervention: No digital mobile intervention applied (Accelerometer was used as outcome objective measure) |
| Coughlin SS, Thind H, Liu B, Wilson LC. Towards research-tested smartphone applications for preventing breast cancer. Mhealth. 2016 Jun;2:26. doi: 10.21037/mhealth.2016.06.02. Epub 2016 Jun 21. PMID: 27390745; PMCID: PMC4933519. | Generic Publication: Opinion Article |
| Currie CL, Larouche R, Voss ML, Higa EK, Spiwak R, Scott D, Tallow T. The impact of eHealth group interventions on the mental, behavioral, and physical health of adults: a systematic review protocol. Syst Rev. 2020 Sep 23;9(1):217. doi: 10.1186/s13643-020-01479-3. PMID: 32967717; PMCID: PMC7513289. | Protocol / Participants: generic on adults +18. Midlife women % has not yet established perhaps <20% |
| Cussler EC, Teixeira PJ, Going SB, Houtkooper LB, Metcalfe LL, Blew RM, Ricketts JR, Lohman J, Stanford VA, Lohman TG. Maintenance of weight loss in overweight middle-aged women through the Internet. Obesity (Silver Spring). 2008 May;16(5):1052-60. doi: 10.1038/oby.2008.19. Epub 2008 Feb 28. PMID: 18309301. | Intervention: Weight Maintenance Website (Doesn’t fall under the working definition of PA mobile /smartphone-based) interventions. |
| Silva, et al (2016). BONE DENSITOMETRY, CTX, LEVEL OF PHYSICAL ACTIVITY, AND STRENGTH IN POSTMENOPAUSAL WOMEN. Artigos Originais Rev Bras Med Esporte. 2016:22 (3). <https://doi.org/10.1590/1517-869220162203150604> | Study Design: Correlation study bone mineral density values (BMD) and level of PA |
| Dallal CM, Brinton LA, Matthews C, et al. Abstract 2519: Is accelerometer-measured physical activity associated with urinary estrogens and estrogen metabolites among postmenopausal women?. Cancer Research. 2013:73(8 Supplement):2519-2519. Doi: 10.1158/1538-7445.AM2013-2519 | Study Design: Correlation study / Outcome measured: urinary estrogens and estrogen metabolites (EM) / Intervention: No Mobile PA intervention applied (Accelerometer was used as an outcome measure not intervention tool. |
| David P, Buckworth J, Pennell ML, Katz ML, DeGraffinreid CR, Paskett ED. A walking intervention for postmenopausal women using mobile phones and Interactive Voice Response. J Telemed Telecare. 2012 Jan;18(1):20-5. doi: 10.1258/jtt.2011.110311. Epub 2011 Nov 3. PMID: 22052963; PMCID: PMC3817710. | Intervention: Traditional calls/ messaging. Doesn't fall under the definition of mHealth smart-phone intervention?/ Outcome: No measurements of menopasual symptomes and PA changes from baseline to end of intervention. |
| de Roon M, van Gemert WA, Peeters PH, Schuit AJ, Monninkhof EM. Long-term effects of a weight loss intervention with or without exercise component in postmenopausal women: A randomized trial. Prev Med Rep. 2016 Dec 9;5:118-123. doi: 10.1016/j.pmedr.2016.12.006. PMID: 27981025; PMCID: PMC5156605. | Intervention: No digital mobile intervention applied - supervised exercise program (Accelerometer was used as outcome objective measure) |
| Diniz TA, Neves LM, Rossi FE, Fortaleza AC, Rosa CS, Tebar WR, Christofaro DG, Freitas-Junior IF. Weekly time spent in the standing position is independently related to a better quality of life in postmenopausal women. Eur J Obstet Gynecol Reprod Biol. 2016 Apr;199:150-5. doi: 10.1016/j.ejogrb.2016.02.011. Epub 2016 Feb 21. PMID: 26930043. | Study Design: Association studystanding time and QoL / Intervention: No mobile PA intervention applied |
| Galiano-Castillo N, Arroyo-Morales M, Lozano-Lozano M, Fernández-Lao C, Martín-Martín L, Del-Moral-Ávila R, Cantarero-Villanueva I. Effect of an Internet-based telehealth system on functional capacity and cognition in breast cancer survivors: a secondary analysis of a randomized controlled trial. Support Care Cancer. 2017 Nov;25(11):3551-3559. doi: 10.1007/s00520-017-3782-9. Epub 2017 Jun 22. PMID: 28639097. | Intervention: web-based exercise program |
| Giannini, et al. Web-based applications to promote women's Health. Maturitas. 2019: 124:148. DOI: <https://doi.org/10.1016/j.maturitas.2019.04.111> | Publication: Conference Proceedings |
| Gibson C, Matthews K, Thurston R. Daily physical activity and hot flashes in the Study of Women's Health Across the Nation (SWAN) Flashes Study. Fertil Steril. 2014 Apr;101(4):1110-6. doi: 10.1016/j.fertnstert.2013.12.029. Epub 2014 Feb 1. PMID: 24491454; PMCID: PMC3972358. | Study Design: Association bet PA and reporting hot flushes / Intervention: No mobile PA intervention applied |
| Rey, et al. The use of lactate thresholds to individualize accelerometer cut-points in breast cancer and healthy post-menopausal women. Are we sure of doing enough physical activity?. EuroPrevent 2017. Available from: <https://esc365.escardio.org/presentation/150102> | Conference proceeding  Intervention: No mobile PA intervention involved / Publication: Conference Proceedings |
| Grossman JA, Arigo D, Bachman JL. Meaningful weight loss in obese postmenopausal women: a pilot study of high-intensity interval training and wearable technology. Menopause. 2018 Apr;25(4):465-470. doi: 10.1097/GME.0000000000001013. PMID: 29088015. | Control: received active wearble intervention (fitbit) - Compare Hgih and Low intensity Trainings |
| Guérin E, Biagé A, Goldfield G, Prud'homme D. Physical activity and perceptions of stress during the menopause transition: A longitudinal study. J Health Psychol. 2019 May;24(6):799-811. doi: 10.1177/1359105316683787. Epub 2017 Jan 5. PMID: 28810371. | Intervention: No digital mobile intervention applied |
| Hardcastle SJ, Jiménez-Castuera R, Maxwell-Smith C, Bulsara MK, Hince D. Fitbit wear-time and patterns of activity in cancer survivors throughout a physical activity intervention and follow-up: Exploratory analysis from a randomised controlled trial. PLoS One. 2020 Oct 19;15(10):e0240967. doi: 10.1371/journal.pone.0240967. PMID: 33075100; PMCID: PMC7571692. | Participants: Men and Women of colorectal cancer (average age 65 older adluts and 59% female/ Outcome: No reproting of menopausal symptoms / primary outcome: engagement with fitbit |
| Im EO, Kim S, Lee C, Chee E, Mao JJ, Chee W. Decreasing menopausal symptoms of Asian American breast cancer survivors through a technology-based information and coaching/support program. Menopause. 2019 Apr;26(4):373-382. doi: 10.1097/GME.0000000000001249. PMID: 30461556; PMCID: PMC6435386 | Intervention: Technology-based coaching intervention, doesn't fall under PA mHealth intervention |
| Nikkhah P. Walking with pedometer, Depression, General health in postmenopausal women. Iranian Registry of Clinical Trials (IRCT2014020616501N1). 2014. Available from: <https://en.irct.ir/trial/15424> | Intervention: Pedometer-based walking. Doesn't fall under the definition of smart-phone intervention |
| Irct- Dastyar (2020). The effect of menopausal self-care application on the severity of menopausal symptoms in postmenopausal women: Designing a menopausal self‐care application and examining its effect on the severity of menopausal symptoms in postmenopausal women referring to health centers in Jiroft in 2020. | Publication: Contacted the corresponding author |
| Kamalnathan P, Aishwarya A. Effect of Telemonitoring Versus Conventional Physiotherapy Techniques in Improving Grip Strength among Postmenopausal Home Makers. Indian Journal of Public Health Research & Development. 2019:10 (8) p427-433. 7p. | Publication: Not found/ Study Design: Poor quality / Irrelavant outcome measured: no PA level (MVPA) instead change in hand strength |
| Kishida, M., & Elavsky, S. (2017). A daily process approach to depict satisfaction with life during the menopausal transition: Physical (in)activity, symptoms, and neuroticism. *Journal of Happiness Studies: An Interdisciplinary Forum on Subjective Well-Being, 18*(3), 631–645. [https://doi.org/10.1007/s10902-016-9743-z](https://psycnet.apa.org/doi/10.1007/s10902-016-9743-z) | Intervention: Doesn't fall under the definition of smart-phone (mHealth) intervention. Accelerometer was used as objective measure. Participants wore an accelerometer for the objective assessment of PA and completed daily Internet surveys at the end of their day. / Study Aim: To examine the between- and within-person association of PA and satisfaction with life (SWL)Multivarient analyses. - Irrelevant outcome |
| Llanos AA, Krok JL, Peng J, Pennell ML, Vitolins MZ, Degraffinreid CR, Paskett ED. Effects of a walking intervention using mobile technology and interactive voice response on serum adipokines among postmenopausal women at increased breast cancer risk. Horm Cancer. 2014 Apr;5(2):98-103. doi: 10.1007/s12672-013-0168-4. Epub 2014 Jan 17. PMID: 24435584; PMCID: PMC3976681. | Outcome: Irrelevant. Women at risk of Breast Cancer |
| Lynch BM, Nguyen NH, Reeves MM, Moore MM, Rosenberg DE, Wheeler MJ, Boyle T, Vallance JK, Friedenreich CM, English DR. Study design and methods for the ACTIVity And TEchnology (ACTIVATE) trial. Contemp Clin Trials. 2018 Jan;64:112-117. doi: 10.1016/j.cct.2017.10.015. Epub 2017 Oct 31. PMID: 29097298. | Publication: Protocol |
| Lyons EJ, Baranowski T, Basen-Engquist KM, Lewis ZH, Swartz MC, Jennings K, Volpi E. Testing the effects of narrative and play on physical activity among breast cancer survivors using mobile apps: study protocol for a randomized controlled trial. BMC Cancer. 2016 Mar 9;16:202. doi: 10.1186/s12885-016-2244-y. PMID: 26960972; PMCID: PMC4784467. | Control: Both groups received mHealth apps with different features - No full text available |
| Manikowska F, Hojan K, Chen PJ, Jóźwiak M, Jóźwiak A. The gait pattern in post-menopausal women. Pilot study. Ortop Traumatol Rehabil. 2013 Nov-Dec;15(6):575-83. doi: 10.5604/15093492.1091513. PMID: 24662904. | Intervention: No digital mobile intervention applied/ Outcome of interest: No PA or menopausal symptoms reproted / Full text in Polish |
| Mattioli AV, Ballerini Puviani M. A Comment on Griffin et al "My Quest, an Intervention Using Text Messaging to Improve Dietary and Physical Activity Behaviors and Promote Weight Loss in Low-Income Women". J Nutr Educ Behav. 2018 Jul-Aug;50(7):754. doi: 10.1016/j.jneb.2018.01.018. PMID: 30047483. | Publication: Letter to the Editor / Intervention: No mobile app identified / Not targeting menopausal women |
| McNeil J, Brenner DR, Stone CR, O'Reilly R, Ruan Y, Vallance JK, Courneya KS, Thorpe KE, Klein DJ, Friedenreich CM. Activity Tracker to Prescribe Various Exercise Intensities in Breast Cancer Survivors. Med Sci Sports Exerc. 2019 May;51(5):930-940. doi: 10.1249/MSS.0000000000001890. PMID: 30694978. | Control: wrist-worn (fitbit) - Compare Hgih and Low intensity Trainings / Intervention: testing lower and higher intensity exercise using Polar A360® activity trackers / No reproting of menopausal symptoms / After Data extraction, it seems that the study was mainly aimed at comparing the differences bet low and intensity exercise using WT among BCS. Irrelevant outcomes measured. |
| McNeil J, Fahim M, Stone CR, O'Reilly R, Courneya KS, Friedenreich CM. Adherence to a lower versus higher intensity physical activity intervention in the Breast Cancer & Physical Activity Level (BC-PAL) Trial. J Cancer Surviv. 2022 Apr;16(2):353-365. doi: 10.1007/s11764-021-01030-w. Epub 2021 Mar 22. PMID: 33754246. | - Both groups received Polar A360® trackers and were included in this analysis (n=30). Comparative group: active intervention |
| Najafabadi, Tadayon M, Abedi P, Farshadbakht F. Impact of pedometer-based walking on menopausal women's sleep quality: a randomized controlled trial. Climacteric. 2016 Aug;19(4):364-8. doi: 10.3109/13697137.2015.1123240. Epub 2016 Jan 12. Erratum in: Climacteric. 2016 Aug;19(4):i. PMID: 26757356. | Intervention: Pedometer-based intervention: doesn't fall under mHealth intervention/ Outcome measured: Sleep disturbance as common menopausal symptoms |
| Catrine Tudor-Locke. WalkMore Walking Interventions for Overweight/Obese Postmenopausal Women. <https://www.clinicaltrials.gov/ct2/show/NCT01519583> | Publication : protocol / Intervention: Pedometer-based intervention:  Registered clinical trial |
| Fergus (2015). Online vs In-Person Lifestyle Intervention for Weight Management and Improved Quality of Life in Breast Cancer Survivors. NCT02861703 https://clinicaltrials.gov/show/NCT02861703, 2015 | Participants: adults 21+ (not targeting menopausal symptoms) / Intervention: Online group-based lifestyle intervention / Publication: Study Protocol (No full text found)  Cochrane Central Register of Controlled Trials |
| Lyons EJ, Baranowski T, Basen-Engquist KM, Lewis ZH, Swartz MC, Jennings K, Volpi E. Testing the effects of narrative and play on physical activity among breast cancer survivors using mobile apps: study protocol for a randomized controlled trial. BMC Cancer. 2016 Mar 9;16:202. doi: 10.1186/s12885-016-2244-y. PMID: 26960972; PMCID: PMC4784467. | Duplicate publication |
| Fetter C, Boll L. (2021). Inspiratory Muscle Training X CardioBreath App Effects On Vagal Modulation And Pulse Wave Velocity. ClinicalTrials.gov Identifier: NCT03672760 <https://clinicaltrials.gov/ct2/show/NCT03672760> | Registered clinical trial  Outcome: Irrelevant. Not looking into any menopausal symptoms or MVPA (PA outcomes) |
| Pelotas Nct (2020) Adaptations to Breast Cancer and Exercise Using Telehealth (ABRACE: telehealth ) ClinicalTrials.gov Identifier: NCT04641377 <https://clinicaltrials.gov/ct2/show/NCT04641377> | Registered clinical trial  Participants: Clinical adult popluation not targeting menopausal women) / Intervention: telehealth (video calls) doesn't fall under the definition of mHealth. Publication: Study Protocol (No full text found) |
| Jyu-Lin Chen (2020). SCOPE-Chinese Women Study (SCOPE). ClinicalTrials.gov Identifier: NCT04326660 https://clinicaltrials.gov/ct2/show/NCT04326660 | Registered clinical trial  Participants: premenopaual (not experiencing any menopausal symptoms) |
| Nguyen NH, Hadgraft NT, Moore MM, Rosenberg DE, Lynch C, Reeves MM, Lynch BM. A qualitative evaluation of breast cancer survivors' acceptance of and preferences for consumer wearable technology activity trackers. Support Care Cancer. 2017 Nov;25(11):3375-3384. doi: 10.1007/s00520-017-3756-y. Epub 2017 May 24. PMID: 28540402. | Duplicate |
| Oppezzo M, Tremmel J, Desai M, Baiocchi M, Ramo D, Cullen M, Prochaska JJ. Twitter-Based Social Support Added to Fitbit Self-Monitoring for Decreasing Sedentary Behavior: Protocol for a Randomized Controlled Pilot Trial With Female Patients From a Women's Heart Clinic. JMIR Res Protoc. 2020 Dec 4;9(12):e20926. doi: 10.2196/20926. PMID: 33275104; PMCID: PMC7748950. | Protocol / Control: Active component (Fitbit) / The tested intervention of social media twitter component doesn't support the SR objectives. |
| Pal, S., Cheng, C. & Ho, S. The effect of two different health messages on physical activity levels and health in sedentary overweight, middle-aged women. BMC Public Health 11, 204 (2011). https://doi.org/10.1186/1471-2458-11-204 | Intervention: Doesn't fall under the definition of smart-phone (mHealth) intervention. The tested intervention was health message |
| Park MJ, Kim HS. Evaluation of mobile phone and Internet intervention on waist circumference and blood pressure in post-menopausal women with abdominal obesity. Int J Med Inform. 2012 Jun;81(6):388-94. doi: 10.1016/j.ijmedinf.2011.12.011. Epub 2012 Jan 21. PMID: 22265810. | Intervention: SMS traditional interventions/ Outcome: Irrelevant not reproting menopausal symptoms or MVPA |
| Ravn Jakobsen P, Hermann AP, Søndergaard J, Wiil UK, Clemensen J. Development of an mHealth Application for Women Newly Diagnosed with Osteoporosis without Preceding Fractures: A Participatory Design Approach. Int J Environ Res Public Health. 2018 Feb 13;15(2):330. doi: 10.3390/ijerph15020330. PMID: 29438343; PMCID: PMC5858399. | Interventon: app designed to provide treatment and medication managment, no PA |
| Reddy (2017). Studying the impact of exercise on hot flashes using mobile exercise tracker, MENQOL scale and hot flash diary. ClinicalTrials.gov Identifier: NCT03236896 <https://www.clinicaltrials.gov/ct2/show/NCT03236896> | Publication: Conference Abstract - Contacted the corresponding author |
| Rocha J, Paxman JR, Dalton CF, Hopkins M, Broom DR. An acute bout of cycling does not induce compensatory responses in pre-menopausal women not using hormonal contraceptives. Appetite. 2018 Sep 1;128:87-94. doi: 10.1016/j.appet.2018.05.143. Epub 2018 May 26. PMID: 29807126. | Participant: premenopausal / Intervention: No mHealth intervention applied / Outcome: irrelevant |
| Alonso-Domínguez R, Sánchez-Aguadero N, Llamas-Ramos I, Lugones-Sánchez C, González-Sánchez S, Gómez-Marcos MA, García-Ortiz L; Ewomen Investigators. Effect of an intensive intervention on the increase of physical activity and the decrease of sedentary lifestyle in inactive postmenopausal. J Adv Nurs. 2021 Apr;77(4):2064-2072. doi: 10.1111/jan.14737. Epub 2021 Jan 22. PMID: 33481300. | Duplicate |
| Ryan P, Papanek P, Csuka ME, Brown ME, Hopkins S, Lynch S, Scheer V, Schlidt A, Yan K, Simpson P, Hoffman R; Striving to be Strong Team. Background and method of the Striving to be Strong study a RCT testing the efficacy of a m-health self-management intervention. Contemp Clin Trials. 2018 Aug;71:80-87. doi: 10.1016/j.cct.2018.06.006. Epub 2018 Jun 9. PMID: 29894865. | Outcome irrelevant: Bone mineral density |
| Tadayon M, Abedi P, Farshadbakht F. Impact of pedometer-based walking on menopausal women's sleep quality: a randomized controlled trial. Climacteric. 2016 Aug;19(4):364-8. doi: 10.3109/13697137.2015.1123240. Epub 2016 Jan 12. Erratum in: Climacteric. 2016 Aug;19(4):i. PMID: 26757356. | Intervention: Pedometer-based intervention: doesn't fall under mHealth intervention |
| Tudor-Locke C, Swift DL, Schuna JM Jr, Dragg AT, Davis AB, Martin CK, Johnson WD, Church TS. WalkMore: a randomized controlled trial of pedometer-based interventions differing on intensity messages. BMC Public Health. 2014 Feb 15;14:168. doi: 10.1186/1471-2458-14-168. PMID: 24528783; PMCID: PMC3931482. | Duplicate / Intervention: Pedometer-based intervention doesn't fall under mHealth definition |
| Vallance JK, Nguyen NH, Moore MM, Reeves MM, Rosenberg DE, Boyle T, Milton S, Friedenreich CM, English DR, Lynch BM. Effects of the ACTIVity And TEchnology (ACTIVATE) intervention on health-related quality of life and fatigue outcomes in breast cancer survivors. Psychooncology. 2020 Jan;29(1):204-211. doi: 10.1002/pon.5298. Epub 2019 Dec 30. PMID: 31763746. | Duplicate intervention: already included. Outcome: No report of menopausal symptoms - Cancer related outcomes were measured: fatigue and QoL - cancer scales |
| van Cappellen-van Maldegem SJM, Mols F, Horevoorts N, de Kruif A, Buffart LM, Schoormans D, Trompetter H, Beijer S, Ezendam NPM, de Boer M, Winkels R, Kampman E, Schuit J, van de Poll-Franse L, Seidell JC, Hoedjes M; OPTIMUM research team. Towards OPtimal TIming and Method for promoting sUstained adherence to lifestyle and body weight recommendations in postMenopausal breast cancer survivors (the OPTIMUM-study): protocol for a longitudinal mixed-method study. BMC Womens Health. 2021 Jul 6;21(1):268. doi: 10.1186/s12905-021-01406-1. PMID: 34229690; PMCID: PMC8258491. | Publication : protocol (The COVID-19 pandemic has delayed the inclusion of PMBC survivors) / Intervention: Doesn't fall under the definition of mHealth/ Outcome: irrelevant |
| Azuma K, Nojiri T, Kawashima M, Hanai A, Ayaki M, Tsubota K; TRF-Japan Study Group. Possible favorable lifestyle changes owing to the coronavirus disease 2019 (COVID-19) pandemic among middle-aged Japanese women: An ancillary survey of the TRF-Japan study using the original "Taberhythm" smartphone app. PLoS One. 2021 Mar 25;16(3):e0248935. doi: 10.1371/journal.pone.0248935. PMID: 33765024; PMCID: PMC7993768. | Participants: Age group 30 -50 with no reporting of menopausal symptoms. Not considered as midlife women |
| Huberty, J., Ehlers, D.K., Kurka, J. et al. Feasibility of three wearable sensors for 24 hour monitoring in middle-aged women. BMC Women's Health 15, 55 (2015). <https://doi.org/10.1186/s12905-015-0212-3> | Intervention: ActiGraph - Purpose of the study was to assess the feasability of using wearable sensors as 24h objective measurements monitors for middle aged women range 30-60. No PA behaviour change outcomes assessed or reporting of menopausal symptoms. |
| Ehlers DK, Huberty JL, de Vreede GJ. Can an evidence-based book club intervention delivered via a tablet computer improve physical activity in middle-aged women? Telemed J E Health. 2015 Feb;21(2):125-31. doi: 10.1089/tmj.2013.0360. Epub 2014 Dec 19. PMID: 25526014. | Intervention: Telemedicine (Tablet based videoconferencing intervention) |
| Ehlers DK, Huberty JL. Middle-aged women's preferred theory-based features in mobile physical activity applications. J Phys Act Health. 2014 Sep;11(7):1379-85. doi: 10.1123/jpah.2012-0435. Epub 2013 Dec 20. PMID: 24368818. | Average age 40 (SD: 10) |
| Rayward, et al. Efficacy of an m-Health Physical Activity and Sleep Intervention to Improve Sleep Quality in Middle-Aged Adults: The Refresh Study Randomized Controlled Trial. Annals of Behavioral Medicine. 2020: 54(7) 470–483, <https://doi.org/10.1093/abm/kaz064> | Participants: targeted both men and women - 83% were female. No gender-specific sub-analysis |

| Reason for exclusions | # |
| --- | --- |
| Duplicates (intervention / publication) | 8 |
| Participant (Premenopausal / Older adults +65 / studies recruited adults aged 18 and older or mixed gender/ different age range 30-50) | 9 |
| Intervention (Pedometer-based/ telehealth/ supervised group-based program/ ActiGraph accelerometer worn as objective measure outcome / non-PA intervention (coaching/ medication apps) | 33 |
| Control (active) | 5 |
| Outcome (irrelevant / e.g., cancer-specific/ bone density) | 5 |
| Publication (opinion letters/ symposium summaries/ protocols) | 8 |
| Study design (cross-sectional/ correlation studies) | 5 |
|  | **73** |

**
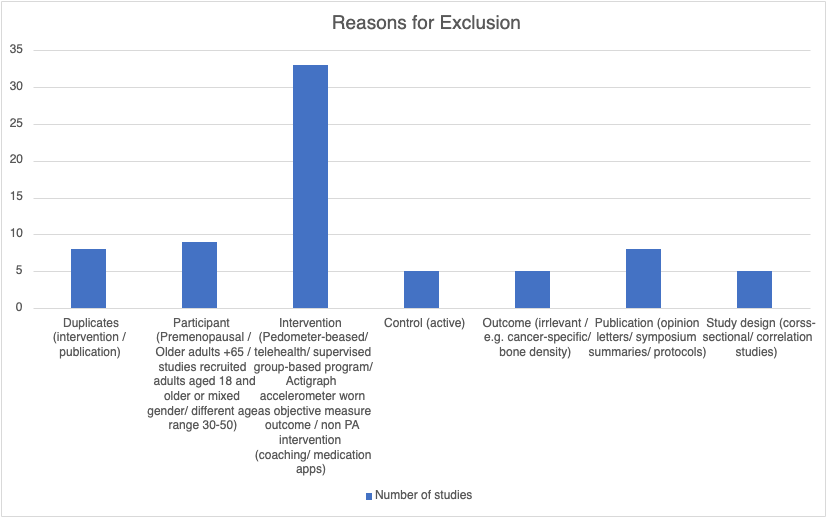
**
