# Supplementary material for: Assessing the Acceptability and Effectiveness of Mobile-Based Physical Activity Interventions for Midlife Women During Menopause: Systematic Review of the Literature
Source: JMIR Mhealth Uhealth. 2022 Dec 9;10(12):e40271. doi: 10.2196/40271 (PMC9789501; doi:10.2196/40271)
Supplement: Multimedia Appendix 3 [file mhealth_v10i12e40271_app3.docx]

**Multimedia Appendix 3: Inter-rater Reliability between independent reviewers**

| Record # from Rayyan full-text | Rater 1 | Rater 2 | Difference |  |  |
| --- | --- | --- | --- | --- | --- |
| 1 | 0 | 0 | 0 |  | Coding Yes=1 |
| 2 | 0 | 0 | 0 |  | Coding No=0 |
| 3 | 0 | 0 | 0 |  |  |
| 4 | 0 | 0 | 0 |  |  |
| 5 | 1 | 1 | 0 |  |  |
| 6 | 0 | 0 | 0 |  |  |
| 7 | 0 | 0 | 0 |  |  |
| 8 | 0 | 0 | 0 |  |  |
| 9 | 0 | 0 | 0 |  |  |
| 10 | 0 | 0 | 0 |  |  |
| 11 | 0 | 0 | 0 |  |  |
| 12 | 1 | 1 | 0 |  |  |
| 13 | 0 | 0 | 0 |  |  |
| 14 | 0 | 0 | 0 |  |  |
| 15 | 0 | 0 | 0 |  |  |
| 16 | 1 | 1 | 0 |  |  |
| 17 | 0 | 0 | 0 |  |  |
| 18 | 1 | 0 | 1 |  |  |
| 19 | 0 | 0 | 0 |  |  |
| 20 | 0 | 0 | 0 |  |  |
| 21 | 0 | 0 | 0 |  |  |
| 22 | 0 | 0 | 0 |  |  |
| 23 | 0 | 0 | 0 |  |  |
| 24 | 1 | 1 | 0 |  |  |
| 25 | 0 | 0 | 0 |  |  |
| 26 | 0 | 0 | 0 |  |  |
| 27 | 1 | 0 | 1 |  |  |
| Number of Zero | | | 25 |  |  |
| Number of Variables | | | 27 |  |  |
| IRR %Agreement | | | 92.59% | Interrater Reliability | 0.92 |
