# Supplementary material for: Assessing the Acceptability and Effectiveness of Mobile-Based Physical Activity Interventions for Midlife Women During Menopause: Systematic Review of the Literature
Source: JMIR Mhealth Uhealth. 2022 Dec 9;10(12):e40271. doi: 10.2196/40271 (PMC9789501; doi:10.2196/40271)
Supplement: Multimedia Appendix 4 [file mhealth_v10i12e40271_app4.docx]

**Multimedia appendix (4)**

Themes and subthemes around acceptability from qualitative included studies

| Acceptability Themes | sub-themes/ Description | Quote(s) from qualitative studies |
| --- | --- | --- |
| Theme1: Perceived usefulness to increase self-awareness of PA and menopause experiences | Mobile apps as a potential solution to raise self-awareness to PA and about monospace experience | *“I was keen to see what does my everyday activity measure and I was sort of quite surprised at how much I actually did [P3, FG1, Age 62, Nguyen, 2017]”.*  *“Definitely you didn’t realise how long you [have been sitting], I think I’ve been sitting for 15 minutes and it’s [actually] an hour, particularly when you’re working, time just flies past and all of a sudden...” [P5, FG2, Age 62, Nguyen, 2017]”.*  *“I relied more on exercise and food intake change, rather than on medical treatments. I tried to start hiking more or walk a lot to make my feeling better [Lee, 2015]”.* |
| Theme2: Perceived Readiness and perceived ease of using mobile apps and WATs | Lack of readiness due to limited technological capabilities and digital literacy  Perceived ease of use of PA mobile technologies: mixed views of using activity trackers and paired apps    Perceived inappropriateness and appearance of wearable PA trackers | *“I am a bit old school I don’t think the experience [using the tracker] would make me necessarily go out and buy one. If I felt I was reasonably active doing what I do, in my normal daily activities, I would probably be happy with that, but then I tend to ignore technology if I can [P7, FG2, Age 62, Nguyen, 2017]”*  *“I had no trouble using them [the trackers] […] all I had to put in my steps […] that was easy [P9, FG3, Age 51, Nguyen, 2017]”*  *“It’s not simple, I agree. It took a few days to get used to the wearable and apps […] [the instruction] quite limited so that took a little bit to set up [P4, FG1, Age 60, Nguyen, 2017]”*  *“Why are you connecting negative experiences of women’s change of life [during menopause], instead of celebrating her change of life? [P3, Session B, Backonja, 2021]”.*  *“I didn’t like wearing it at night. I didn’t feel comfortable. I wanted to be away from all sort of electrical kinds of things when I sleep [Nguyen, 2017]”*  *“There’s no category that says strength training, like sometimes you would use walking to measure strength training [P4, FG1, Age 60, Nguyen, 2017]”.*  *“I thought that was quite good, but it actually wore off after a while. I think you need something to encourage you to do it [exercise] [P2, FG1, Age 60, Nguyen, 2017]”*  *“I would not wear the tracker [Polar A300] going out due to its large size and the way they looked [P2, FG1, Age60, Nguyen, 2017]”*  *“I would buy just for the fact it’s got a bigger screen, easier to see, the numbers are facing you rather than sideways [P5, FG2, Age 62, Nguyen, 2017]”.* |
| Theme 3: Midlife women’s favoured features of PA apps | Step counting: basic feature  Setting goals and monitor progress  Real-time feedback of PA | *“Using the app, I just feel as if I had very limited [skills] ... and it’s probably through my lack of skills... and limited interaction with it” (P7, FG2, Age 62, Nguyen, 2017).*  *“I just followed the step count. I found the step count encouraged me to have to get over my 10,000 steps. […] I think, because that’s a number that just always sticks in my head.” (P9, FG3, Age 51, Nguyen, 2017)*  *“If you get to say 8000 [steps] in a day, you’re more motivated to do those extra 2000 because you’re so close. It’s like Why would I stop now? I might as well keep going [P10, FG3, Age 52, Nguyen, 2017]”*  *“It was a Garmin, say the goal is 10,000 [steps] and you had a lazy morning, it drops down to 8000 and then 6000 and […] No, I still want to do my 10,000 [P1, FG1, Age 57, Nguyen, 2017]”*  *“… I think what you put on the message will be very important. People won’t be encouraged by repetitive text notifications [Lee, 2015]”.*  *“I preferred these [inspiring emails sent by [Fitbit] over not receiving feedback [P6, FG2, Age 61, Nguyen, 2017]”.* |
