# Supplementary material for: Assessing the Acceptability and Effectiveness of Mobile-Based Physical Activity Interventions for Midlife Women During Menopause: Systematic Review of the Literature
Source: JMIR Mhealth Uhealth. 2022 Dec 9;10(12):e40271. doi: 10.2196/40271 (PMC9789501; doi:10.2196/40271)
Supplement: Multimedia Appendix 5 [file mhealth_v10i12e40271_app5.docx]

**Multimedia Appendix 5: BCTs Coding Process**

| **Record# EndNote**    **Author (Year)** | **Description of Mobile Interventions** | **BCTs coding**  **(Reviewer 1)** | **BCTs coding**  **(Reviewer 2)** | **Frequency of BCTs** |
| --- | --- | --- | --- | --- |
| #13    Cadmus-Bertram, et al (2016) | **Fitbit-based PA intervention**   - Fitbit One used as a wearable self-tracker device that clips to waistband or in a pocket. - Participants were encouraged to perform 150 minutes/week of MVPA and walk 10,000 steps/day. Based on ActiGraph baseline data, individualized plans with specific goals were set to participants to achieve. - Dashboard platform: Summary data of PA intensities were displayed on an online dashboard website where users can change goals, view feedback or log PA manually. - Set goals were refined at week 4 using a follow-up call to evaluate progress.     (Original script)    Fitbit-based intervention using Fitbit One (Web-Based Tracking Group): a low-touch, Fitbit-based PA intervention focused on self- monitoring/self-regulation skills.  Fitbit tracker and website: Fitbit One: an accelerometer- based device that clips to the waistband or bra, or is placed in a pocket. Summary data are shown on the tracker’s display and PA intensities and temporal patterns are available on the website. The online “dashboard” to display only PA data. Individualized goals were set for the first 4 weeks of the study (using data observed on the baseline ActiGraph) and the participant committed to a specific plan to achieve these. A follow-up call at 4 Weeks was used to evaluate progress and refine goals. Both groups were asked to perform 150 minutes/week of MVPA and walk 10,000 steps/day. | 1.1 Goal setting (behaviour)  1.4 Action planning   1.5 Review behaviour goals  2.3 Self-monitoring of behaviour   2.2 Feedback on behaviour   2.6 Biofeedback        Control (Traditional Pedometer)  1.1 Goal setting (behaviour)  2.3 Self-monitoring of behaviour “pedometer”  1.2 Problem solving (printed materials”  1.4 Action planning | 1.1 Goal setting (behaviour)  1.4 Action planning   1.5 Review behaviour goals  2.3 Self-monitoring of behaviour   2.2 Feedback on behaviour   2.6 Biofeedback | 6 |
| #10    Butryn, et al (2016) | **Fitbit-based PA, Blended Intervention**  12 in-person, 90-min group-based behavioural modification sessions (during first 3 months), alongside Fitbit® FlexTM sensor to be worn daily to capture users’ PA intensity, step counts, minutes of sleep, calorie intake with personalized goals.  (Original script)    Technology-enhanced lifestyle modification program to achieve and maintain high levels of MVPA.    Blended apporach during Phase I: initial behavior change stage (baseline-3M), and Phase II: maintenance stage(4-6M) with Main componnet was: Technology-enhanced lifestyle modification program: To achieve and maintain high levels of MVPA.    Self-monitoring and social connectivity were facilitated by use of "PA sensors" that were synced to a "web platform".    Details: (IN-PERSON: 12, 90-min group-based behavioural modification sessions were facilitated by ***group leaders*** with prior experience in lifestyle modification with tailored content to address concerns of midlife women and facilitate PA and healthy eating behaviours using behavioral and cognitive skills to increase minutes per week of MVPA and healthy eating. Group leaders provided feedback on progress and encouraged participants to offer each other suggestions. + ONLINE: Fitbit® FlexTM sensor to be worn daily and web platfrom to capture and display detailed information on personalised “dash-board” of participants' PA intensity over the course of the current day, displayed in 15-minute increments, as measured by the Fitbit® FlexTM as well as step totals, minutes of sleep, water and calorie intake, and/or milestones such as reaching 10,000 steps in a day. The web platform also displayed a “Leaderboard” on which participants viewed their PA progress (as measured by the PA Fitbit sensor). Group leaders monitored the Community Boards and replied to participants’ posts peri- odically, typically acknowledging participants’ efforts and providing encouragement.  Monthly step totals for each participant were displayed in rank order (highest on top); rankings by minutes of MVPA were available by changing display options.   Participants were also encouraged to communicate and post once per week via a private message board (“Community Board”). | 1.1 Goal setting (behaviour)  1.2 Problem solving    2.3 Self-monitoring of behaviour   2.5 Monitoring of outcome of behaviour without feedback  1.6 Discrepancy between current behaviour and goal “Leaderboard”  2.2 Feedback on behaviour “Group Leaders monitored the community board”  2.6 Biofeedback    3.1 Social support (unspecified)   6.2 Social comparison   9.1 Credible source “Group Leaders w experience in lifestyle modification” | 1.1 Goal setting (behaviour)  1.2 Problem solving  2.5 Monitoring of outcome of behaviour without feedback  3.1 Social support (unspecified)  9.1 Credible source  2.2 Feedback on behaviour  2.6 Biofeedback  2.3 Self-monitoring of behaviour  6.2 Social comparison  1.6 Discrepancy between current behaviour and goal | 10 |
| #62  Lynch, et a l (2019)      #70  Nguyen, et al (2021) | **Wearable Activity Monitor and App: Garmin (Multi-components mobile based intervention)**   - Wrist-worn Garmin Vivofit 2 activity monitor with Garmin app displaying data on steps, distance, calories and sleep time. Visual prompts to alert inactivity via a red bar and an audible beep. - Participants were provided a one-off behavioral feedback and goal-setting session alongside basic training on how to set up the wearable. - A booklet on the consequences and barriers to PA was shared with a summary of their weekly PA based on baseline. - 5 follow-up counselling calls to facilitate and sustain behaviour change.     (Original script)  3 components delivered: : (1) a wrist-worn Garmin Vivofit2® activity monitor to wear for 12 weeks, (2) behavioral feedback (based on baseline behavioral data) and goal-setting in a single face-to-face session with a Trial team member at Cancer Council Victoria to receive workbook on the consequences of PA and barriers to behaviour change where women encouraged to generate behavior change goals guided by a motivational interviewing approach + basic training for the set up (including downloading and installing the smartphone/tablet/PC application) and use of their wearable technology activity monitor, and (3) five telephone-delivered behavioral counseling sessions delivered by the same Trial team member to facilitate adoption and maintenance of behavior change.    The Garmin Vivofit 2® does not need to be charged (it uses a built in battery lasting 1 year), and it provides inactivity alerts visually via a red bar on the display and an audible beep. The move bar is reset by walking for a few minutes. One limitation of most wearable technologies that provide such inactivity alerts is that they may count standing time as inactive time. Therefore, participants will be counselled to ignore prompts that occur when they are moving or standing but encouraged to take some extra steps to reset the move bar. The device can store movement and sleep data for up to 7 days; these data are uploaded to the Garmin app via Bluetooth.    Participants are helped to install the smartphone/tablet/PC application, and to activate their online account, during the face-to-face session with a trial staff member. Participants are encouraged to access their physical activity and sedentary behaviour data at least weekly via the smartphone/tablet/PC application. ACTIVATE Trial staff can access data from participants' Garmin Vivofit 2® through an API developed and administrated by Garmin. Hence, the research team can monitor compliance with this part of the intervention, and discuss issues of non- wear with participants during the five telephone calls. Telephone-delivered behavioural counselling: a Trial team member telephones each primary intervention group participant on five occasions to facilitate maintenance of behaviour change. The first two calls are made weekly, followed by two calls made a fortnight apart, and a final call one month later. Calls focus on practical and acceptable personal behavioural change initiatives, including how to more effectively use the activity monitor for self- monitoring, identifying environmental cues to reduce and break up sedentary behaviour, setting updated goals and problem-solving to identify the most appropriate and sustainable strategies for the participant. Calls also help troubleshoot any difficulties participants are experiencing with the activity monitor or the associated smartphone/tablet/PC app. | 1.1 Goal setting (behaviour)  1.2 Problem solving    2.3 Self-monitoring of behaviour   1.5 Review behaviour goal “setting updated goals”  2.2 Feedback on behaviour “follow-up calls”  2.6 Biofeedback “Accessed via the Garmin app”  4.1 Instruction on how to perform the behavior   7.1 Prompts/cues” inactivity alerts/ identify environmental cues to reduce sedentariness” | 2.6 Biofeedback  2.3 Self-monitoring of behaviour  1.1 Goal setting (behaviour)  4.1 Instruction on how to perform the behavior  2.2 Feedback on behaviour  5.3 Information about social and environmental consequences  “a booklet on consequences and barriers to PA”  3.1 Social support (unspecified)  “behavioural counselling”  7.1 Prompts/cues  1.2. Problem solving  1.5 Review behaviour goals | 10 |
| #56  Sengupta, et al (2020) | **Smartwatch and smartphone app: HerBeat**  - A wrist-worn smartwatch (Moto 360), and Android app with features of goal setting, track progress, feedback surveys and videos.  - A web-platform was used to monitor users’ data.  (Original script)  The HerBeat: smartwatch and the smartphone (gender-specific mHealth app for secondary prevention of CHD in women) that were provided for the duration of the study. The HerBeat prototype included a wrist-worn smartwatch (Moto 360 2nd Gen, Android Wear OS 2.0) and a smartphone (Samsung Galaxy S6, Android 7.0), with the app installed on both devices and a web-based dashboard for monitoring participant data. The 4 features of the prototype included (1) goal setting, (2) progress, (3) ecological momentary assessment (EMA) surveys, and (4) videos.    The goal setting feature allowed participants to set multiple walking goals for up to 60 min each. Study participants were tasked with setting their own PA goals in terms of the number of minutes walked. Participants were also prompted to report their readiness to begin PA and their current level of energy on a scale of 1 to 10. After setting a PA goal, each participant was sent a motivational message that encouraged exercise. Data about the participant goal setting and subsequent PA performance were monitored through a web-based dashboard in real time by a trained professional. The progress function permitted participants to review the number of minutes walked, number of steps taken, and distance covered in miles. If participants had not completed their goal when seeking progress, they were presented with the number of minutes remaining to goal completion.  If a goal was completed, the participant was sent a gender-specic graphic user interface (GUI) with a congratulatory message for achieving their goal. The EMAs are described in the Measurement section. The nal feature provided participants access to 9 customized short videos, developed by the principal investigator (PI) with expertise in behavioral medicine and women’s CV health, on healthy eating behavior and on guidelines for safe PA. The app also sent two types of behavior change intervention messages. If the participant had not set a PA goal by 4 PM daily, a message prompting them to exercise was sent. If participants were proactive in setting and achieving walking goals, they were sent a positive reinforcing message. | 1.1 Goal setting (behaviour)  1.4 Action planning   2.1 Monitoring of behaviour by others without feedback “participants’ goal setting and PA performance were monitored through by a trained professional”  2.3 Self-monitoring of behaviour    2.6 Biofeedback “presents total minutes remaining to goal completion”  4.1 Instruction on how to perform the behavior   6.1 Demonstration of the behaviour “videos”  7.1 Prompts/cues “motivational messages?”  10.4 Social reward “congratulatory message” | 2.3 Self-monitoring of behaviour    2.6 Biofeedback  1.1 Goal setting  1.4 Action planning  2.1 Monitoring of behaviour by others without feedback  1.6 Discrepancy between current behaviour and goal “If participants had not completed their goal when seeking progress, they were presented with the number of minutes remaining to goal completion.”  10.4 Social reward  5.3 Information about health consequences  7.1 Prompts/cues | 9 |
| #99  Valle, et al (2017) | **Self-weighing and activity tracker mobile intervention**  WELL Body self-regulation (multi-components) intervention  - Educational in-person session about weight gain and its consequences, in addition to the importance of energy balance through diet and PA behaviour change.  - Self-monitoring of weight using a wireless scale that sync data to a companion app (INT and INT+ arms) and PA wearable tracker: Withings Pulse, Cambridge, MA. (Only INT+ arm). Participants taught how to use the scale and access the Withings app.  - Weekly messages on cognitive behavioural management strategies; e.g., problem solving, behaviour monitoring, social support,   - Feedback was tailored to each user in both groups based on objectively synced data.  (Original script)  Both intervention arms: (1) a face-to-face individual session; (2) a Bluetooth and Wifi-enabled wireless scale (Withings WS-30, Cambridge, MA). with access to a companion mobile app and website with graphs of weight trends; (3) 24 weekly email-delivered behavioral lessons; and (4) Tailored feedback on self- weighing and weight data. INT+ arm only: (5) monitor activity daily using activity tracker.    WELL Body self-regulation intervention: 1) face to face session: education about weight gain in BCS, behaviors associated with body weight, and health consequences of weight gain. An overview of energy balance through diet and PA behaviors and the importance of self-weighing as an indicator of energy balance and emphasized daily use of the wireless scale as a tool or indicator of progress with diet and PA behaviors. 2) Self-montoring of weight using a wireless scale that was configured during the initial visit to automatically sync weight data with the individual’s online account and/or mobile app. Both intervention groups were instructed to monitor their weight by weighing themselves daily using the wireless scale and taught how to use the scale and access the Withings website or app for viewing weight trends over time. 3) weekly emails on skills and cognitive behavioral weight control strategies (e.g., self- monitoring, problem solving, finding social support). INT+ arm only: (5) monitor activity daily using activity tracker.    Activity monitoring intervention: The INT+ group received all of the above and were asked to wear an activity tracker (Withings Pulse, Cambridge, MA), which interfaced with the wireless scale and synced data to a single online account. The only difference between INT+ and INT group lessons was in the weekly homework; INT+ participants were encouraged to track their activity daily in addition to weighing themselves daily. Tailored feedback to this group incorporated both objective PA monitoring information garnered from the activity tracker and weight data from the wireless scale. To feedback on weight and daily self-weighing, the message provided specific feedback on whether participants were monitoring their activity and meeting their weekly exercise recommendation. The messages reinforced the importance of monitoring activity, of regular exercise for weight management, or provided specific strategies for adopting and maintaining PA behaviors. | 1.2 Problem solving    2.3 Self-monitoring of behaviour “PA”  2.4 Self-monitoring of outcome of behaviour “Self-weighing”  2.2 Feedback on behaviour “Tailored based on synced data”  2.6 Biofeedback “Access to weight trends on the app”  3.1 Social support (unspecified)  4.1 Instruction on how to perform the behavior  7.1 Prompts/cues | 2.6 Biofeedback  2.3 Self-monitoring of behaviour  12.5 Adding objects to the environment “wireless scale”  3.1 Social support (unspecified)  1.2 Problem solving  2.2 Feedback on behaviour  5.1 Information about health consequences “education about weight gain in BCS, behaviors associated with body weight, and health consequences of weight gain”  8.3 Habit formation “daily weighing”  2.4 Self-monitoring of outcome of behaviour  4.1 Instruction on how to perform the behavior  7.1 Prompts/cues  1.6 Discrepancy between current behaviour and goal “specific feedback on whether participants were monitoring their activity and meeting their weekly exercise recommendation” | 11 |
| #101  Joseph, et al (2021) | **Smart Walk Smartphone App + Fitbit wearable**   - Smart Walk app designed to deliver culturally-appropriate, educational and behavioural PA promotion materials, and facilitate building an online supporting community for PA. - Self-monitoring and tracking app features were integrated with a wrist-worn Fitbit activity wearable to allow users track the MVPA progress. - The app allows users to record PA manually for stationary cycling, water aerobics and swimming activities     (Original script)   Smart Walk smartphone app and text messages. Features available on the Smart Walk app include personal profile pages, multimedia PA promotion modules, discussion board forums, and an activity tracking feature that integrates with Fitbit activity monitors. The behavioral PA goal of the intervention was for participants to meet national guidelines of 150 minutes per week of at least moderate-intensity PA, with walking emphasized as the primary behavior to achieve this goal. In addition to: SMS text messaging: 3 PA promotional text messages each week.    Smart Walk Smartphone App: 4 main features designed to promote daily PA: (1) personalized profile pages: provided participants with a platform to share select biographical information (ie, picture name, age, neighborhood or local area of residence, and brief biographical narrative) with other study participants. Profiles were designed to help facilitate building of a web-based community and social support network for PA. (2) Multimedia PA promotion modules delivered on a weekly basis in the form of brief videos and electronic text with images: were the primary delivery channels for the educational and behavioral components of the program. These modules consisted of text- and image-based PA promotion materials. (3) Discussion boards: to discuss the weekly PA modules and give or receive social support + Community Board forum and a Meet-up forum, where participants could share information and/or discuss generic topics and (4) a PA self-monitoring or tracking tool: that integrates with Fitbit (Fitbit Inc) activity monitors. received a wrist-worn Fitbit Inspire HR (Fitbit Inc) activity monitor to wear throughout the study. Data collected from the Fitbit integrates with the Smart Walk app to allow participants to view and track the minutes of MVPA performed during the study. For activities not recorded by the activity monitor (eg, stationary cycling, water aerobics, and swimming), participants could manually enter the activity via the app’s tracking feature.    Cultural characteristic was addressed in the intervention (content): for instance, highlighting that "portrayed regular PA as a key behavior to help participants perform their caretaking, familial, and community responsibilities with more energy and for a longer duration throughout the life span." and "informed participants that engaging in PA at the levels recommended by the study (ie, 150 min per week) will not substantially change their body shape unless they also change their dietary habits." | 1.1 Goal setting (behaviour)  1.2 Problem solving   2.3 Self-monitoring of behaviour “Fitbit Inc integrated Smart Walk App”  2.6 Biofeedback    3.1 Social support (Unspecified) “Discussion board forums for social networking”  4.1 Instruction on how to perform the behaviour   6.1 Demonstration of the behaviour “Multimedia PA promotion videos” | 2.3 Self-monitoring of behaviour  2.6 Biofeedback  1.1 Goal setting (behaviour)  1.2 Problem solving  3.1 Social support (Unspecified)  5.3 Information about social and environmental consequences “portrayed regular PA as a key behavior to help participants perform their caretaking, familial, and community responsibilities with more energy and for a longer duration throughout the life span”  4.1 Instruction on how to perform the behaviour   6.1 Demonstration of the behaviour | 7 |
| #108  Kashfi, et al (2021) | **WhatsApp-based mobile intervention**   - A WhatsApp group was created to deliver educational and motivational messages about PA and its importance during menopause. - 3 educational materials (written, clips or pamphlets) were sent every week for (10 weeks). The platform was used to answer any quires about menopausal symptoms and PA. - Participants were also encouraged to perform a specific exercise program to at least engage in 30 minutes of brisk walking, 5 days a week during the period of intervention (1month).   (Original script)   An educational, motivational WhatsApp group based mobile intervention created and, every week, three educational written messages, clips, or pamphlets about physical activity and its importance during menopause were sent for 10 weeks.  Educational package on PA: (1) the importance and benefits of doing physical activity in maintaining the health of menopause women, (2) Motivational messages on using the stairs instead of the elevator, walking along the path back to your home instead of using a taxi, parking the car away from the destination, hiking, walking with family members after dinner, washing the car by yourself instead of going to the car wash, doing the house chores such as vacuuming the floor, washing windows, gardening by the person, and walking to work, extracted from the book by Allameh, 26 was provided and confirmed by the authorities. Then, we created a WhatsApp group named “Health of Dashti Women”. The WhatsApp group was formed and managed by the researcher. Every week, three educational written messages, clips, or pamphlets about physical activity and its importance during menopause extracted from the educational package were sent to the group members for 10 weeks. The exercise program offered to the EG group to encourage users to at least engage in 30 minutes of fast walking with moderate intensity, at least 5 days a week for one month. Besides, answering any questions about menopause and its symptoms and ways to reduce complications and PA. | 1.1 Goal setting (behaviour)  1.2 Problem solving “discussion on menopausal symptoms and PA”  1.4 Action planning “30 min of brisk walking, 5 days/week for 1 month”  6.1 Demonstration of the behaviour “exercise program Clips?” | 5.1 Information about health consequences  4.1 Instruction on how to perform the behaviour  3.1 Social support unspecified  6.1 Demonstration of the behaviour  1.1 Goal setting (behaviour)  1.2 Problem solving  1.4 Action planning  7.1 Prompts/cues “3 educational written messages sent in group?” | 8 |
| #21  Nguyen, et al (2017) | **Wearable Activity Trackers (WATs):** Fitbit One, Jawbone Up 24, Garmin Vivofit 2, Garmin Vivosmart, Garmin Vivoactive and Polar A300 and their apps   - Commercially available activity trackers with step-count and non-movement notification features. - Each woman was randomly assigned to two or three of the six WATs, each device to be worn for 2 weeks with a non-wear wash out period. - Users were advised on how to set up trackers, sync devices with apps, and about main features (step counts, sitting alert)     (Original script)    6 Commercially available PA WATs in Austrailia that have two basic features in order to be included in this focus group study: a step-count (pedometer) function and a non-movement notification. Apps inclusion crtiteria include but not limited to: user friendly (based on users reviews), affordability (less than $500AUD, which excluded the Apple Watch). Fitbit One, Jawbone Up24, Garmin Vivofit2, Garmin Vivosmart, Garmin Vivoactive and Polar A300. Participants were also briefly guided on how to operate the tracker (push button or swipe), its basic features (step counts, sitting alert) and its monitor app and were encouraged to sync the tracker with the app daily. | 2.3 Self-monitoring of behaviour   2.6 Biofeedback    4.1 Instruction on how to perform the behaviour   7.1 Prompts /cues “non-movement alerts” | 2.3 Self-monitoring of behaviour   2.6 Biofeedback    4.1 Instruction on how to perform the behaviour  7.1 Prompts /cues | **4** |
| #103  Kim (2020) | **Living lab for a mobile-based health**   - LLm Health: is a culturally sensitive mobile intervention based on Fitbit devices, the mobile app, and weekly self-efficacy SMS. - The app was designed to offer 6 features: a step counter, an exercise timer, an online chat function, health information (e.g., menopausal symptoms, stress management, weight control), level of cardiovascular risk, and overall health status dashboard. The group chat feature was linked to an SNS to encourage information sharing and increase emotional and network support. - To encourage adherence and self-monitoring of walking, (1) the app was synchronized with the Fitbit Alta for automatic data transmission of total steps and moderate-intensity exercise time. Users were also able to input their strength exercise and stretching time. (2) Step goals were set 3 times every 4 weeks, and (3) A reward of a medal image was sent via SMS upon reaching the goal. - The app was designed to send a self-efficacy SMS once a week to highlight barriers to and benefits of exercise.     (Original script)    The LLm Health program comprised a 24-week walking program using Fitbit devices, the mobile app, and social cognitive interventions.  The app’s functions include: a step counter, an exercise timer, an online chat function, health information, level of cardiovascular risk, and health status.  The mobile app was designed to measure the number of steps and exercise duration of users, using smart bands, and we attempted to encourage users to self-monitor their walking adherence through the development of a mobile app linked with the smart bands (Fitbit Alta).  During the 12-week adaptation period, the following interventions were applied to strengthen the social psychological factors affecting the change in health behavior and social-cognitive capabilities (self-efficacy, social support, etc) of the enhanced treatment group: sending SMS to improve self-efficacy, setting exercise goals, providing feedback, and social networking service (SNS) interaction. In addition, a photovoice activity was planned to represent the exercise promotion factors of community and the sense of local community that KC participants perceive.     _ To encourage participation in the study, we set up automatic transmission of Fitbit Alta data and app synchronization notifications once a day on the registered participants’ mobile phones. The daily number of steps and moderate-intensity exercise time were obtained via synchronization with the Fitbit Alta, and participants were able to input their strength exercise and stretching time during the day.   _The app had 6 main functions, all listed on the main screen: number of walking steps, duration of exercise, chatting, health information, cardiovascular disease risk, and health status.  _The health information covered 12 topics, including cardiovascular disease, musculoskeletal disease, menopausal symptoms, aging prevention, stress management, pharmacy and hospital available on weekends, weight control, healthy eating, stretching, strength exercise, and cancer screening for women.  _ The group chat feature of the app was linked to an SNS (eg, KaKao talk) to encourage information sharing among participants, leading to an increase in emotional and network support.  To encourage exercise adherence during the 12-week adaptation period, step goals were set 3 times every 4 weeks, and encouragement with a medal image was sent via SMS whenever participants reached the goal. The app was designed to send a self-efficacy SMS once a week, so that participants could overcome the barriers to exercise and recognize the benefits of exercise.  A total of 12 self-efficacy SMS messages were designed, all with less than 60 characters that focused on mood, lack of time, lack of interest, physical discomfort, lack of social support for exercise, limitations because of appearance, limitations of place, fatigue, lack of exercise skills, negative perception of migrant worker exercise, and lack of awareness regarding the benefits of exercise. | 1.1 Goal setting (behaviour) “walking”  1.2 Problem solving “Self-efficacy SMS”  1.5 Review behaviour goal “step goals were set 3 times every 4 weeks”  2.3 Self-monitoring of behaviour   2.6 Biofeedback “Fitbit Alta”  3.1 Social support (Unspecified) “Group chat feature”  6.1 Demonstration of the behaviour “photovoice exercise feature”  10.4 Social reward “Medal image upon reaching the goal”  4.1 Instruction on how to perform the behaviour  7.1 Prompts/cues “notifications once a day”  5.1 Information about health consequences  3.3 Social support emotional “encourage information sharing and increase emotional and network support”  15.1 Verbal persuasion about capability | 2.3 Self-monitoring of behaviour   2.6 Biofeedback  3.3 Social support emotional “encourage information sharing and increase emotional and network support”  15.1 Verbal persuasion about capability  1.2 Problem solving  1.1 Goal setting (behaviour)  1.5 Review behaviour goal(s)  10.4 Social reward  3.1 Social support (Unspecified)  5.1 Information about health consequences  6.1 Demonstration of the behaviour  4.1 Instruction on how to perform the behaviour  7.1 Prompts/cues “notifications once a day” | 13 |

*** Data Extraction and Quality Assessment templates can be available upon request.**
